# Trifluoromethylthiolation Carbonylation of Unactivated Alkenes via Distal Migration

**DOI:** 10.1021/acs.orglett.4c04151

**Published:** 2024-11-20

**Authors:** Ren-Guan Miao, Yuanrui Wang, Zhi-Peng Bao, Xiao-Feng Wu

**Affiliations:** † Dalian National Laboratory for Clean Energy, Dalian Institute of Chemical Physics, Chinese Academy of Sciences, Dalian, Liaoning 116023, China; ‡ Leibniz-Institut für Katalyse e.V., 18059 Rostock, Germany

## Abstract

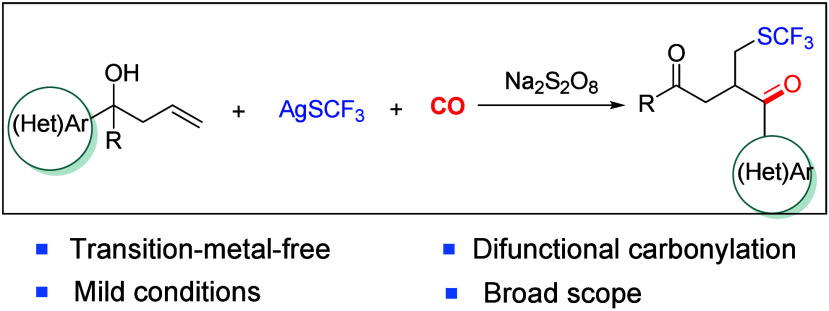

Sulfur-containing compounds represent a significant category of organic compounds, and the introduction of sulfur groups into organic compounds can effectively enhance their biological activity and synthetic diversity. Although a variety of difunctionalization reactions of alkenes based on sulfur radicals have been documented, significant challenges remain in the carbonylative difunctionalization of unactivated alkenes by the addition of a sulfur radical. Herein, we present a trifluoromethylthiolative carbonylation reaction of unactivated alkenes, which goes through the addition of a trifluoromethylthiol radical to unactivated alkenes and then carbonylation of the newly generated carbon radical intermediate. A heterocyclic/aryl migration in the presence of carbon monoxide is crucial for the success of this methodology and finally resulted in the formation of sulfur-containing carbonylated products in good yields.

Sulfur-containing compounds represent a significant class of organic compounds that are pervasively present in a multitude of pharmaceuticals, bioactive molecules, and functional materials.^1^ The incorporation of sulfur-containing groups into organic compounds can markedly enhance their biological activity and synthetic diversity.^2^ Consequently, the construction of the C–S bond has emerged as a prominent area of research, giving rise to a plethora of synthetic methodologies. Among them, sulfur radicals serve as highly efficient radical centers for the synthesis of sulfur-containing compounds. On the other hand, unactivated alkenes represent a significant category of chemical raw materials, offering a diverse range of applications in chemical synthesis and the construction of numerous other molecular entities. The difunctionalization of unactivated alkenes at the C=C double bond position is a particularly intriguing phenomenon.^3^ Consequently, the difunctionalization of alkenes by the addition of sulfur radicals represents an effective approach for the introduction of sulfur groups. Up to now, the difunctionalization of alkenes with a variety of sulfur radical sources, including sulfonyl chlorides, sulfonyl hydrazides, thiols, thiophenols, disulfides, and silver trifluoromethylthiol, has been reported (Scheme 1A).^4−9^

**Scheme 1:**
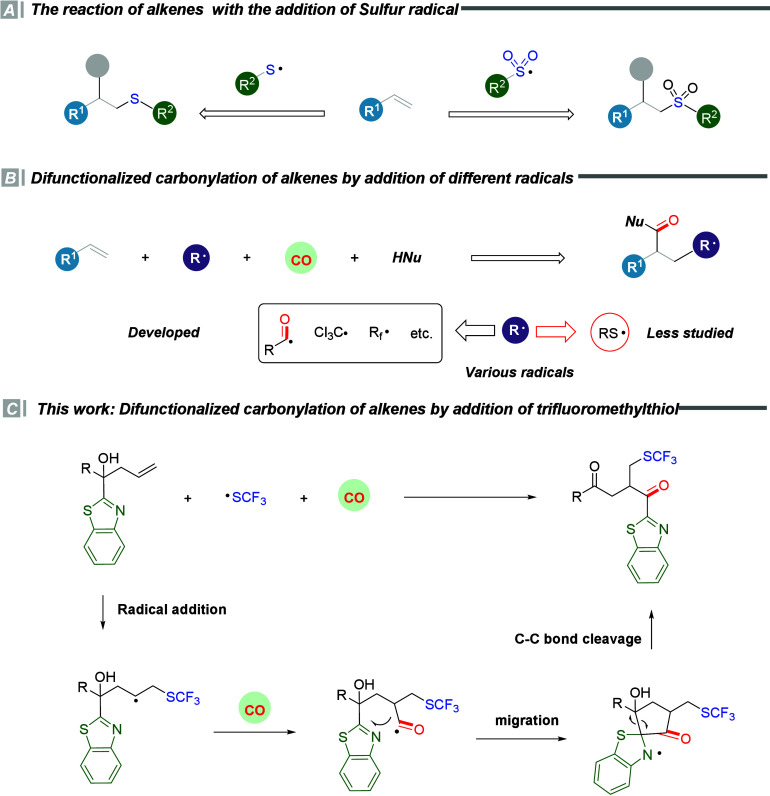
Difunctionalization of Alkenes via Radical Addition

Carbon monoxide (CO) is a cost-effective and readily accessible C1 source. Carbonylated compounds can be synthesized easily through the incorporation of carbon monoxide (CO).^10^ In recent years, the rapid development of transition metal catalysis and visible light catalysis has led to a proliferation of activation and transformation methods for carbon monoxide (CO). Significant advancements have been made in the field of constructing carbonyl-containing compounds through carbonylation procedures, particularly in the introduction of two functional groups to alkenes in a selective and controlled manner through carbonylation. Recently, numerous difunctional carbonylation reactions of unactivated alkenes by carbon radical addition have been documented, including those with acyl radicals, trichloromethyl radicals, trifluoromethyl radicals, and polyfluoroalkyl radicals (Scheme 1B).^11−15^ However, due to the high activity of sulfur radicals, the carbon radicals generated after adding olefins are more readily quenched by different nucleophiles. Consequently, the carbonylative double functionalization of unactivated alkenes with the addition of sulfur radical represents a significant challenge in chemical research.

The reported examples revealed that the presence of an electron-withdrawing group on the generated carbon radical is conducive to the radical addition of unactivated alkenes and then carbonylative difunctionalization. Hence, we hypothesized that a sulfur radical with electron-withdrawing groups could solve the problem of carbonylative difunctionalization of unactivated alkenes. We learned that AgSCF_3_ can be readily oxidized to generate a trifluoromethylsulfenyl radical, which can participate in addition reactions of alkenes. Therefore, AgSCF_3_ was chosen as the sulfur radical source to afford the difunctionalization carbonylation reaction of unactivated alkenes. We hypothesized that the initial step involves the generation of the trifluoromethylthiol radical, which then undergoes addition to the unactivated alkenes and gives a new carbon radical. Subsequently, the generated carbon radical captures carbon monoxide, forming an acyl radical. Ultimately, the target compound will be generated through migration rearrangement (Scheme 1C).

Based on the aforementioned design, we initially selected alkene (**1a**), containing allylic, benzothiazole, and tertiary alcohol groups, along with 1.5 equiv of AgSCF_3_ (**2a**) as the template substrates using K_2_S_2_O_8_ (3 equiv) as the oxidant in DMSO to establish the best reaction conditions (Table 1). At the beginning, a 29% yield of the target product was formed under 60 bar of CO at 30 °C (Table 1, entry 1). In the screening of different solvents, decreased yield was obtained, and DMSO was found to be the optimal solvent for this reaction (Table 1, entries 2 and 3). Subsequently, Na_2_S_2_O_8_ and (NH_4_)_2_S_2_O_8_ were tested as oxidants, and the yield could reach 66% when Na_2_S_2_O_8_ was employed as the oxidant (Table 1, entries 4 and5). Through modification of the quantity of oxidant (2.0 equiv), the yield of the desired product can be further improved to 71% (Table 1, entry 6). Furthermore, reducing the amount of AgSCF_3_ to 1.0 equiv was attempted, and a decreased yield of 61% was observed (Table 1, entry 7). Subsequently, an attempt was made to reduce the amount of CO to 40 bar, which resulted in a decreased yield of 59% (Table 1, entry 8). Reducing the reaction time to 12 h resulted in a decreased yield of 60% due to the decreased conversion of substrate (Table 1, entry 9). No target product could be detected in the absence of an oxidant (Table 1, entry 10). Following the screening of various conditions, it was determined that **1a** (0.1 mmol), **2a** (0.15 mmol), Na_2_S_2_O_8_ (2 equiv), DMSO (1 mL), and 60 bar of CO at 30 °C represented the optimal reaction conditions, resulting in an isolated yield of 71%. It is worth mentioning that benzo[*d*]thiazol-2-yl(phenyl)methanone as the main byproduct could be detected during the optimization process.

**Table 1:**
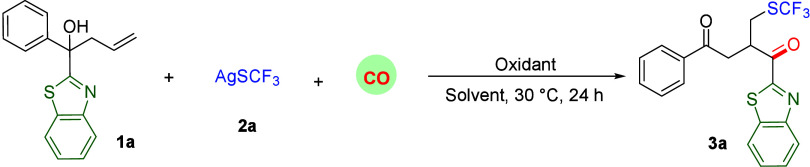
Optimization of Reaction Conditions^a^

entry	oxidant	solv.	yield (%)
1	K_2_S_2_O_8_ (3 equiv)	DMSO	29
2	K_2_S_2_O_8_ (3 equiv)	DMF	trace
3	K_2_S_2_O_8_ (3 equiv)	CH_3_CN	N.D.
4	Na_2_S_2_O_8_ (3 equiv)	DMSO	66
5	(NH_4_)_2_S_2_O_8_ (3 equiv)	DMSO	42
6	Na_2_S_2_O_8_ (2 equiv)	DMSO	71
7^b^	Na_2_S_2_O_8_ (2 equiv)	DMSO	61
8^c^	Na_2_S_2_O_8_ (2 equiv)	DMSO	59
9^d^	Na_2_S_2_O_8_ (2 equiv)	DMSO	60
10		DMSO	N.D.

a Reaction conditions: **1a** (0.1 mmol), **2a** (0.15 mmol), oxidant (3.0 equiv), CO (60 bar), DMSO (1.0 mL), 30 °C, 24 h, isolated yields. N.D.: not detected.

b **2a** (1.0 equiv).

c CO (40 bar).

d 12 h.

Once the optimal reaction conditions had been determined, a series of substrate testing was carried out (Table 2). The high reactivity of heterocyclic rings results in a higher migration rate of benzothiazole in the reaction compared to that observed with aromatic rings. Initially, electron-donating groups substituted at the *para* position of the aromatic ring were tested, including methyl, isopropyl, *tert*-butyl, and phenoxy. The reaction yielded the target products (**3b**–**3e**) in moderate yields, in general. Furthermore, methoxy, methyl, and bromine substitutions at the *meta* position of the aromatic ring were investigated, and it was observed that both electron-withdrawing groups and electron-donating groups could produce moderate yields of the desired products (**3f**–**3h**). Furthermore, methyl substitution at the *ortho* position on the aromatic group was investigated. It was observed that the greater the steric hindrance, the lower the yield (**3i**). Furthermore, substrates with disubstituted aromatic rings were tested, and moderate yields were observed (**3j**, **3k**). It is noteworthy that the greatest compatibility is observed when the substituents on the aromatic group are dielectron-withdrawing groups. In addition to benzothiazole, other heterocyclic rings were also investigated, and it was found that all of them could be transformed to the target products in moderate to good yields (**3l**–**3n**). Additionally, the scope of the diaryl substrates was investigated, and the target products (**3o**, **3p**) were obtained in a straightforward manner. Moreover, alkyl and aryl substrates were also tested, and it was found that they could also afford the target compounds, albeit in relatively low yields (**3q**–**3s**). Finally, some substrates were identified as having low reactivity, resulting in the production of only a limited quantity of the corresponding products (**3t**–**3v**), and no reaction occurred when PhSH or PhSSPh was tested as the substrate. For the homoallyl propanol backbone part, no desired product could be detected when there was one carbon more or less.

**Table 2:**
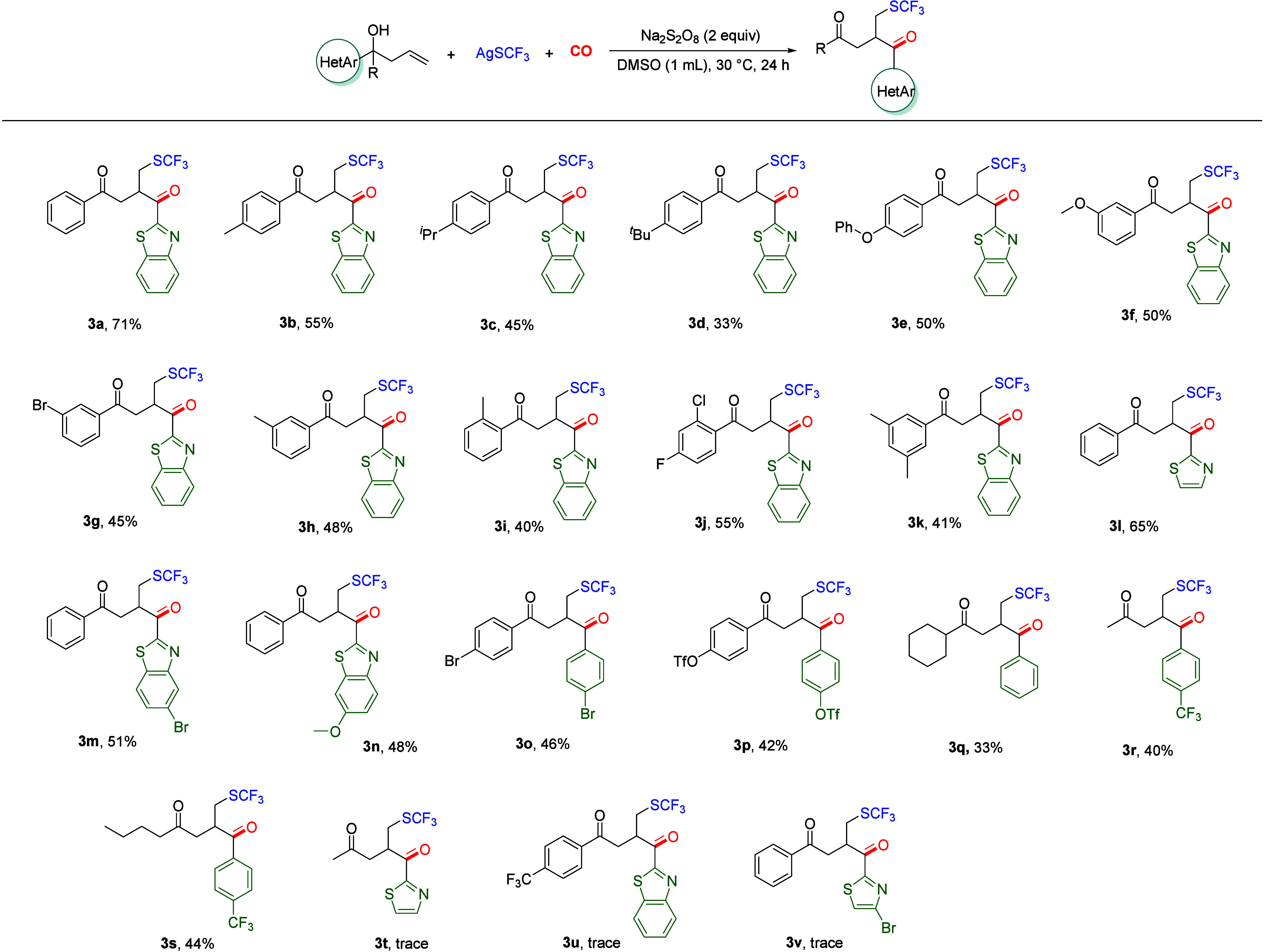
Substrate Scope of Unactivated Alkenes^a^

a Reaction conditions: **1a** (0.1 mmol), **2a** (0.15 mmol), Na_2_S_2_O_8_ (2 equiv), CO (60 bar), DMSO (1.0 mL), 30 °C, 24 h, isolated yields.

In order to gain some insight into the reaction pathway, control experiments were conducted, and the results are presented in Scheme 2. We added TEMPO and BHT under the standard conditions, and no target product could be generated, which indicates that the reaction likely involved radical intermediates. However, no radical-captured intermediates were detected, which might be due to the low stability of the formed compounds (Scheme 2A). Furthermore, an attempt was made to scale up the reaction to a 2 mmol scale under the standard conditions, and a 55% yield of the desired product was obtained without any problem (Scheme 2B). Additionally, the further cyclization reaction of the produced product with Lawesson’s reagent was also realized, and 56% yield of the thiophene derivative **4a** can be produced easily from product **3a** (Scheme 2C).

**Scheme 2:**
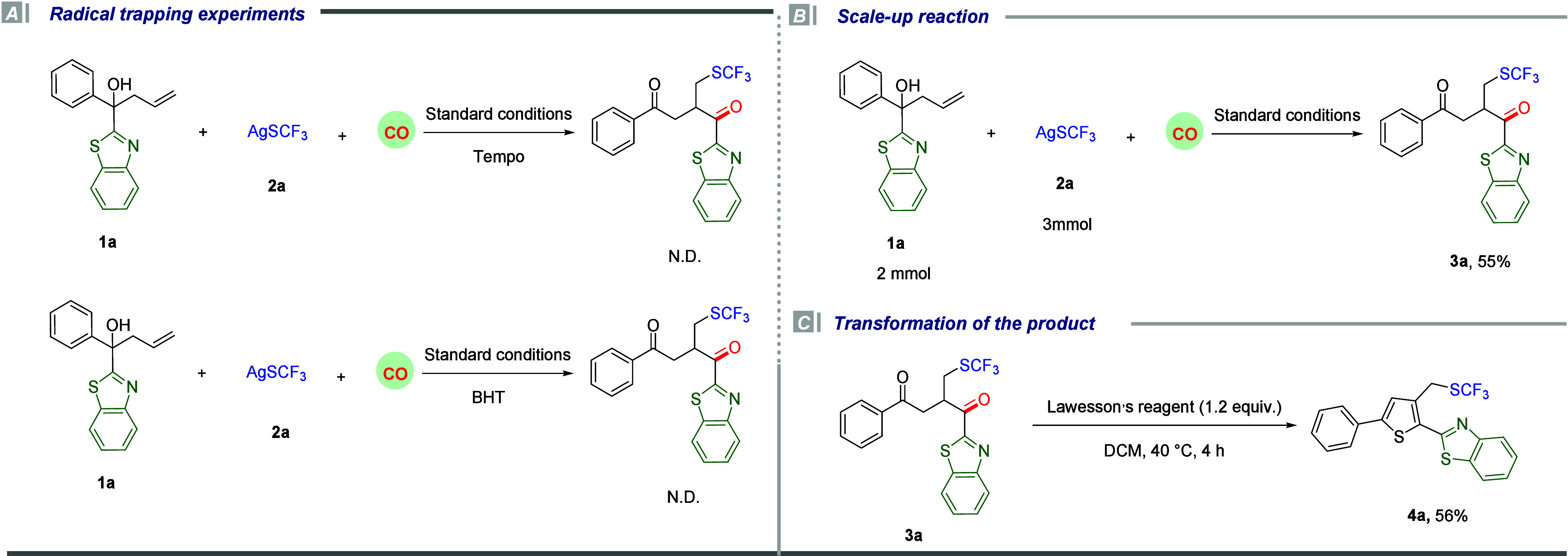
Control Experiments and Synthetic Applications

Based on the above experiments and previously reported reactions, we proposed a possible reaction pathway (Scheme 3). Initially, AgSCF_3_ generates sulfur radical **A** through the action of an oxidant. Subsequently, the sulfur radical reacts with alkene to form intermediate **B**. With the participation of CO, the carbon radical captures CO to generate intermediate **C**. Thereafter, the generated acyl radical attacks the heterocycle to obtain intermediate **E**. Ultimately, intermediate **E** is oxidized and deprotonated by an oxidant to yield target product **3a**.

**Scheme 3:**
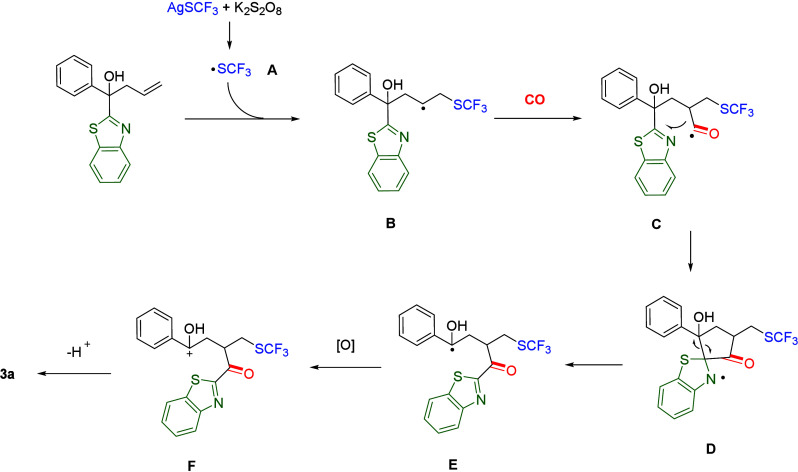
Proposed Mechanism

In summary, we have developed a new difunctional carbonylation reaction of unactivated alkenes with the participation of the sulfur radical and carbon monoxide. In this reaction, a trifluoromethylsulfur radical was generated through the oxidation of AgSCF_3_, and a variety of sulfur-containing 1,4-diketone skeleton compounds were successfully obtained through intramolecular migration with the participation of CO. This reaction also broadens the potential applications of difunctional carbonylation of alkenes, offering the possibility of realizing intermolecular reactions involving sulfur radical addition to alkenes. Additionally, it was discovered that the product could be subjected to further conversion to thiophene derivatives.

## Data Availability

The data underlying this study are available in the published article and its Supporting Information.

## Supporting Information Available

General comments, general procedure, analytical data, and NMR spectra (PDF)
