# Supplementary material for: Trifluoromethylthiolation Carbonylation of Unactivated Alkenes via Distal Migration
Source: Org Lett. 2024 Nov 20;26(47):10189–94. doi: 10.1021/acs.orglett.4c04151 (PMC11613685; doi:10.1021/acs.orglett.4c04151)
Supplement: Supplementary file 1 — ol4c04151_si_001.pdf [file ol4c04151_si_001.pdf]

Supporting Information

Trifluoromethylthiolation carbonylation of unactivated alkenes via distal migration

Ren-Guan Miao,[a,b] Yuanrui Wang,[a] Zhi-Peng Bao,[a,b] and Xiao-Feng Wu[a.b]*

a. Dalian National Laboratory for Clean Energy, Dalian Institute of Chemical Physics, Chinese Academy of Sciences, 116023 Dalian, Liaoning, China, E-mail: xwu2020@dicp.ac.cn

b. Leibniz-Institut für Katalyse e.V., 18059 Rostock, Germany

Table of Contents

Supporting Information 1

1. General Information 1

2. General Procedure 1

- **2.1 Synthesis of starting materials** 1
- **2.2 General procedure for heteroaryl migration.** 2

3. Control Experiments 3

4. Transformation of the product 4

5. Characterization Data of the Corresponding Product 5

6. The NMR Spectrum 17

1. General Information

Unless otherwise noted, all reactions were carried out under N_2. All reagents were from commercial sources and used as received without further purification. All solvents were dried by standard techniques and distilled prior to use. Column chromatography was performed on silica gel (200-300 meshes) using petroleum ether (bp. 60~90 °C) as eluent. ^{1}H and ^{13}C NMR spectra were taken on 400 MHz or 700 MH)z instruments and spectral data were reported in ppm relative to tetramethylsilane (TMS) as the internal standard and $CDCl_3$ (^{1}H NMR δ 7.26, ^{13}C NMR δ 77.16) as solvent. All coupling constants (J) are reported in Hz with the following abbreviations: s = singlet, d = doublet, dd = double doublet, ddd = double doublet of doublets, t = triplet, dt = double triplet, q = quartet, m = multiplet, br = broad. All reactions were monitored by GC-FID or NMR analysis. HRMS data was obtained with Micromass HPLC-Q-TOF mass spectrometer (ESI-TOF) or Agilent 6540 Accurate-MS spectrometer (Q-TOF).

Safety Statement: Because of the high toxicity of carbon monoxide, all the reactions should be performed in an autoclave. The laboratory should be well-equipped with a CO detector and alarm system.

2. General Procedure

2.1 Synthesis of starting materials

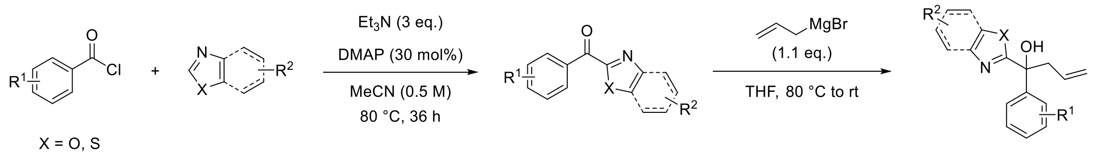

Step I: To a solution of 4-dimethylaminopyridine (DMAP, 146 mg, 1.2 mmol) in acetonitrile (4.0 mL) was sequentially added acyl chloride (0.76 g, 4.0 mmol), thiazole (0.14 mL, 2.0 mmol), and triethylamine (0.84 mL, 6.0 mL) at room temperature. The resulting mixture was heated at 80 °C for 36 h. After cooled to room

temperature, the reaction was quenched with sat. aq. NH_4Cl, and the product was extracted with ethyl acetate. The combined organic layer was washed with brine, dried over Na_2SO_4 and concentrated under reduced pressure. The crude mixture was purified by silica gel column chromatography (dichloromethane/petroleum ether = 1/5 to 1/1) to afford **1** as white or yellow solid.

Step II: To an oven-dried 25 mL double neck round bottom flask, allyl magnesium bromide (1.1 equiv) was added dropwise to a solution of ketone 1 (1.0 equiv) in dry THF (0.2 M) under N_2 atmosphere at 0 °C. The resulting mixture was warmed gradually to room temperature and stirred for 0.5-2 h. After completion of the reaction, the reaction mixture was quenched with a saturated NH_4Cl solution, extracted with ethyl acetate and dried before purified on a silica column using an eluent of ethyl acetate/petroleum ether (1/20) to afford **2** as white or yellow solid.

2.2 General procedure for heteroaryl migration.

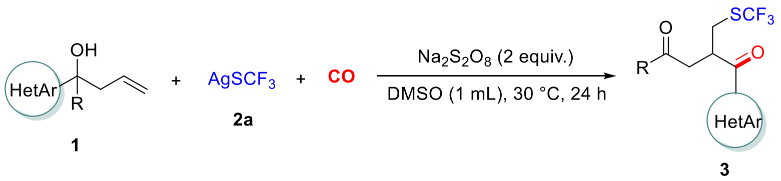

A 4 mL screw-cap vial was charged with **1** (0.1 mmol; 1 equiv), **2a** (0.15 mmol; 1.5 equiv; 31.2 mg), $Na_2S_2O_8$ (0.2 mmol; 2 equiv; 47.6 mg) and an oven-dried stirring bar. The vial was closed with a Teflon septum and cap and connected to the atmosphere via a needle. Then DMSO (1 mL) was added with a syringe under N_2 atmosphere. The closed autoclave was flushed two times with nitrogen (~ 10 bar), and a pressure of 60 bar CO were charged. The autoclave was then placed on a magnetic stirrer. The reaction mixture was stirred at 30 °C for 24 h. After the reaction, the pressure was released carefully. The mixture was concentrated under vacuum. The crude product was purified by column chromatography (PE/EA = 20/1) on silica gel to afford the corresponding products.

2 mmol scale reaction:

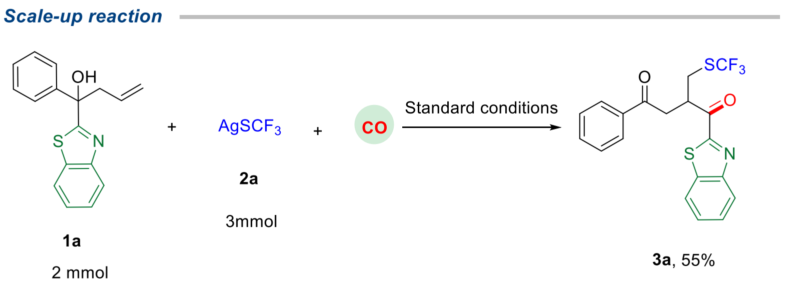

A 50 mL round bottom flask was charged with **1a** (2 mmol; 562 mg), **2a** (3 mmol; 1.5 equiv; 624 mg), $Na_2S_2O_8$ (4 mmol; 2 equiv; 952 mg), and an oven-dried stirring bar. The vial was closed with a Teflon septum and cap and connected to the atmosphere via a needle. Then DMSO (10 mL) was added with a syringe under N_2 atmosphere. The closed autoclave was flushed two times with nitrogen (~ 10 bar), and a pressure of 60 bar CO were charged. The autoclave was then placed on a magnetic stirrer. The reaction mixture was stirred at 30 °C for 24 h. After the reaction, the pressure was released carefully. The mixture was concentrated under vacuum. The crude product was purified by column chromatography (PE/EA = 20/1) on silica gel to afford the corresponding product **3a** (55%; 449.9 mg).

3. Control Experiments

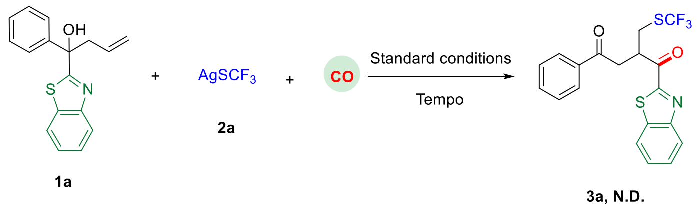

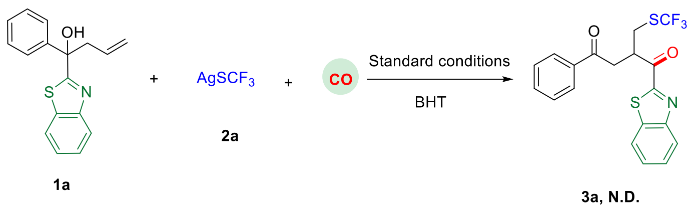

A 4 mL screw-cap vial was charged with **1a** (0.1 mmol), **2a** (0.2 mmol), $Na_2S_2O_8$ (2 equiv.), radical scavenger (3 equiv.) and an oven-dried stirring bar. The vial was closed with a Teflon septum and cap and connected to the atmosphere via a needle.

Then DMSO (1 mL) was added with a syringe under N_2 atmosphere. The closed autoclave was flushed two times with nitrogen (~ 10 bar), and a pressure of 60 bar CO were charged. The autoclave was then placed on a magnetic stirrer. The reaction mixture was stirred at 30 °C for 24 h.

4. Transformation of the product

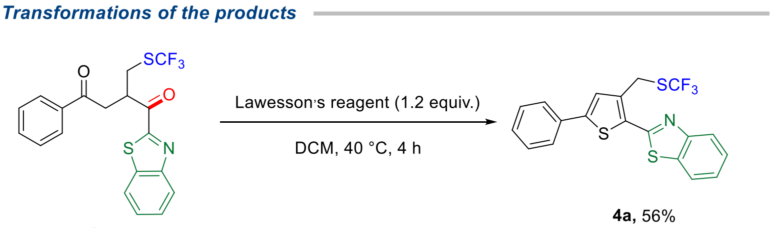

3a **4a**, 56%

A 4 mL screw-cap vial was charged with **3a** (0.2 mmol), Lawesson's reagent (1.2 equiv.) and DCM (4 mL). The reaction mixture was stirred at 40°C for 4 h. After the reaction, the pressure was released carefully. The mixture was concentrated under vacuum. The crude product was purified by column chromatography (PE/EA = 20/1) on silica gel to afford the corresponding product **4a** (56%).

5. Characterization Data of the Corresponding Product

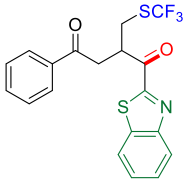

1-(benzo[*d*]thiazol-2-yl)-4-phenyl-2-(((trifluoromethyl)thio)methyl)butane-1,4-dione

The reaction solution was processed as general experimental procedure to afford the corresponding product **3a** as a yellow oil (29.0 mg, 71%; PE/EA = 20/1).

^{1}H NMR (400 MHz, $CDCl_3$) δ 8.26 – 8.17 (m, 1H), 8.05 – 7.91 (m, 3H), 7.60 – 7.52 (m, 3H), 7.46 (t, J = 7.7 Hz, 2H), 5.05 – 4.64 (m, 1H), 3.86 (dd, J = 18.0, 8.2 Hz, 1H), 3.67 – 3.54 (m, 2H), 3.30 (dd, J = 13.7, 7.2 Hz, 1H).

^{13}C NMR (101 MHz, $CDCl_3$) δ 196.9, 194.6, 165.0, 153.6, 137.6, 136.0, 133.7,130.8 (q, J_{C-F} = 306.6 Hz), 128.8, 128.3, 128.1, 127.2, 125.9, 122.5, 42.6, 40.0, 30.6 (q, J_{C-F} = 2.1 Hz).

^{19}F NMR (376 MHz, $CDCl_3$) δ -41.2.

HRMS (ESI-TOF) m/z: Calcd for $[M+H]^+$ $C_{19}H_{15}F_3NO_2S_2$ 410.0491; Found: 410.0492.

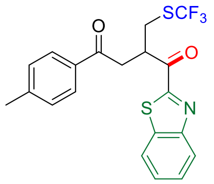

1-(benzo[*d*]thiazol-2-yl)-4-(*p*-tolyl)-2-(((trifluoromethyl)thio)methyl)butane-1,4-dione

The reaction solution was processed as general experimental procedure to afford the corresponding product **3b** as a yellow oil (23.3 mg, 55%; PE/EA = 20/1).

^{1}H NMR (400 MHz, $CDCl_3$) δ 8.25 – 8.17 (m, 1H), 8.05 – 7.96 (m, 1H), 7.88 (d, J = 8.2 Hz, 2H), 7.66 – 7.50 (m, 2H), 7.29 – 7.26 (m, 2H), 4.87 – 4.69 (m, 1H), 3.83 (dd, J = 17.9, 8.1 Hz, 1H), 3.65 – 3.52 (m, 2H), 3.29 (dd, J = 13.6, 7.1 Hz, 1H), 2.42 (s,

3H).

^{13}C NMR (101 MHz, $CDCl_3$) δ 196.5, 194.8, 165.1, 153.6, 144.7, 137.6, 133.6, 130.8 (q, $J_{C\text{-}F}$ = 306.6 Hz), 129.5, 128.4, 128.1, 127.2, 122.5, 42.6, 40.1, 30.6 (q, $J_{C\text{-}F}$ = 2.1 Hz), 21.8.

^{19}F NMR (376 MHz, $CDCl_3$) δ -41.3.

HRMS (ESI-TOF) m/z: Calcd for $[M+H]^+$ $C_{20}H_{17}F_3NO_2S_2$ 424.0647; Found: 424.0653.

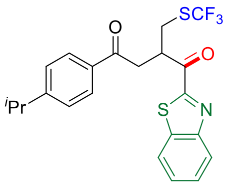

1-(benzo[*d*]thiazol-2-yl)-4-(4-isopropylphenyl)-2-(((trifluoromethyl)thio)methyl)butane-1,4-dione

The reaction solution was processed as general experimental procedure to afford the corresponding product **3c** as a yellow oil (20.3 mg, 45%; PE/EA = 20/1).

^{1}H NMR (400 MHz, $CDCl_3$) δ 8.20 (dd, J = 7.4, 1.4 Hz, 1H), 8.04 – 7.96 (m, 1H), 7.91 (d, J = 8.4 Hz, 2H), 7.63 – 7.50 (m, 2H), 7.32 (d, J = 8.3 Hz, 2H), 4.86 – 4.65 (m, 1H), 3.82 (dd, J = 17.9, 8.0 Hz, 1H), 3.64 – 3.52 (m, 2H), 3.28 (dd, J = 13.6, 7.1 Hz, 1H), 3.00 – 2.93 (dt, J = 13.8, 6.9 Hz, 1H), 1.26 (d, J = 6.9 Hz, 6H).

^{13}C NMR (101 MHz, $CDCl_3$) δ 196.5, 194.8, 165.1, 155.4, 153.7, 137.6, 134.0, 130.8 (q, $J_{C\text{-}F}$ = 306.4 Hz), 128.6, 128.1, 127.2, 127.0, 126.0, 122.6, 42.7, 40.1, 34.4, 30.7 (q, $J_{C\text{-}F}$ = 2.2 Hz), 23.8.

^{19}F NMR (376 MHz, $CDCl_3$) δ -41.3.

HRMS (ESI-TOF) m/z: Calcd for $[M+H]^+$ $C_{22}H_{21}F_3NO_2S_2$ 452.0960; Found: 452.0964.

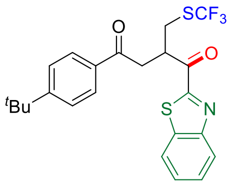

1-(benzo[*d*]thiazol-2-yl)-4-(4-(*tert*-butyl)phenyl)-2-(((trifluoromethyl)thio)methyl)butane-1,4-dione

The reaction solution was processed as general experimental procedure to afford the corresponding product **3d** as a yellow oil (15.3 mg, 33%; PE/EA = 20/1).

^{1}H NMR (400 MHz, CDCl$_3$) δ 8.23 – 8.17 (m, 1H), 8.04 – 7.96 (m, 1H), 7.95 – 7.88 (m, 2H), 7.63 – 7.51 (m, 2H), 7.50 – 7.44 (m, 2H), 4.98 – 4.49 (m, 1H), 3.83 (dd, J = 17.9, 8.0 Hz, 1H), 3.66 – 3.52 (m, 2H), 3.29 (dd, J = 13.6, 7.1 Hz, 1H), 1.34 (s, 9H).

^{13}C NMR (101 MHz, CDCl$_3$) δ 196.5, 194.7, 165.1, 157.6, 153.6, 137.6, 135.4, 133.6, 130.8 (q, J_{C-F} = 306.6 Hz), 128.0, 127.2, 126.0, 125.8, 122.5, 42.7, 40.1, 35.3, 31.2, 30.6 (q, J_{C-F} = 2.0 Hz).

^{19}F NMR (376 MHz, CDCl$_3$) δ -41.3.

HRMS (ESI-TOF) m/z: Calcd for $[M+H]^+$ $C_{23}H_{23}F_3NO_2S_2$ 466.1117; Found: 466.1127.

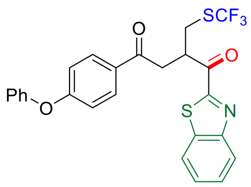

1-(benzo[*d*]thiazol-2-yl)-4-(4-phenoxyphenyl)-2-(((trifluoromethyl)thio)methyl)butane-1,4-dione

The reaction solution was processed as general experimental procedure to afford the corresponding product **3e** as a yellow oil (25.1 mg, 50%; PE/EA = 20/1).

^{1}H NMR (400 MHz, CDCl$_3$) δ 8.25 – 8.17 (m, 1H), 8.02 – 7.92 (m, 3H), 7.63 – 7.50 (m, 2H), 7.44 – 7.36 (m, 2H), 7.25 – 7.17 (m, 1H), 7.11 – 7.05 (m, 2H), 7.04 – 6.96 (m, 2H), 4.85 – 4.71 (m, 1H), 3.81 (dd, J = 17.9, 8.2 Hz, 1H), 3.66 – 3.48 (m, 2H), 3.28 (dd, J = 13.7, 7.2 Hz, 1H).

^{13}C NMR (101 MHz, CDCl$_3$) δ 195.4, 194.7, 165.0, 162.6, 155.4, 153.6, 137.6, 130.8 (q, J_{C-F} = 306.5 Hz), 130.6, 130.2, 128.1, 127.2, 125.9, 124.9, 122.6, 120.4, 117.4, 42.7, 39.9, 30.6 (q, J_{C-F} = 2.0 Hz).

^{19}F NMR (376 MHz, CDCl$_3$) δ -41.2.

HRMS (ESI-TOF) m/z: Calcd for $[M+H]^+$ $C_{25}H_{19}F_3NO_3S_2$ 502.0753; Found: 502.0757.

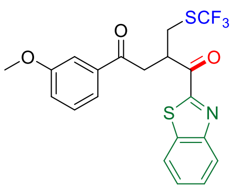

1-(benzo[*d*]thiazol-2-yl)-4-(4-methoxyphenyl)-2-(((trifluoromethyl)thio)methyl)butane-1,4-dione

The reaction solution was processed as general experimental procedure to afford the corresponding product **3f** as a yellow oil (22.0 mg, 50%; PE/EA = 20/1).

^{1}H NMR (700 MHz, $CDCl_3$) δ 8.21 (d, J = 8.1 Hz, 1H), 8.00 (d, J = 7.8 Hz, 1H), 7.63 – 7.52 (m, 3H), 7.48 (dd, J = 2.2, 1.7 Hz, 1H), 7.38 (t, J = 8.0 Hz, 1H), 7.16 – 7.10 (m, 1H), 4.85 – 4.72 (m, 1H), 3.86 – 3.81 (m, 4H), 3.62 (dd, J = 18.0, 5.4 Hz, 1H), 3.55 (dd, J = 13.8, 6.3 Hz, 1H), 3.28 (dd, J = 13.8, 7.2 Hz, 1H).

^{13}C NMR (176 MHz, CDCl3) δ 196.8, 194.7, 165.0, 160.0, 153.6, 137.6, 137.4, 130.8 (q, $J_{C\text{-}F}$ = 306.6 Hz), 129.8, 128.1, 127.2, 126.0, 122.6, 121.0, 120.4, 112.4, 55.6, 42.7, 40.3, 30.6 (q, $J_{C\text{-}F}$ = 2.0 Hz).

^{19}F NMR (376 MHz, $CDCl_3$) δ -41.3.

HRMS (ESI-TOF) m/z: Calcd for $[M+H]^+$ $C_{20}H_{17}F_3NO_3S_2$ 440.0596; Found: 440.0604.

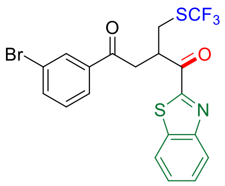

1-(benzo[*d*]thiazol-2-yl)-4-(3-bromophenyl)-2-(((trifluoromethyl)thio)methyl)butane-1,4-dione

The reaction solution was processed as general experimental procedure to afford the corresponding product **3g** as a yellow oil (21.9 mg, 45%; PE/EA = 20/1).

^{1}H NMR (400 MHz, $CDCl_3$) δ 8.21 (d, J = 7.9 Hz, 1H), 8.10 (s, 1H), 8.00 (d, J = 8.0 Hz, 1H), 7.90 (d, J = 7.8 Hz, 1H), 7.71 (d, J = 8.0 Hz, 1H), 7.63 – 7.52 (m, 2H), 7.36 (t, J = 7.9 Hz, 1H), 4.83 – 4.73 (m, 1H), 3.81 (dd, J = 18.0, 8.1 Hz, 1H), 3.62 – 3.50 (m, 2H), 3.28 (dd, J = 13.7, 7.3 Hz, 1H).

^{13}C NMR (101 MHz, CDCl$_3$) δ 195.7, 194.4, 164.8, 153.6, 137.8, 137.6, 136.6, 131.4, 130.8 (q, J_{C-F} = 306.6 Hz), 130.5, 128.2, 127.3, 126.9, 126.0, 123.2, 122.6, 42.7, 40.0, 30.6 (q, J_{C-F} = 2.2 Hz).

^{19}F NMR (376 MHz, CDCl$_3$) δ -41.2.

HRMS (ESI-TOF) m/z: Calcd for $[M+H]^+$ $C_{19}H_{14}BrF_3NO_2S_2$ 487.9596; Found: 487.9603.

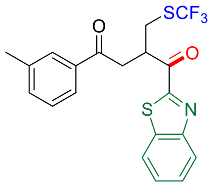

1-(benzo[*d*]thiazol-2-yl)-4-(*m*-tolyl)-2-(((trifluoromethyl)thio)methyl)butane-1,4-dione

The reaction solution was processed as general experimental procedure to afford the corresponding product **3h** as a yellow oil (20.3 mg, 48%; PE/EA = 20/1).

^{1}H NMR (400 MHz, CDCl$_3$) δ 8.21 (dd, J = 7.4, 1.5 Hz, 1H), 8.03 – 7.96 (m, 1H), 7.77 (dd, J = 4.2, 1.5 Hz, 2H), 7.66 – 7.51 (m, 2H), 7.42 – 7.30 (m, 2H), 4.87 – 4.64 (m, 1H), 3.83 (dd, J = 18.0, 8.0 Hz, 1H), 3.64 – 3.52 (m, 2H), 3.28 (dd, J = 13.7, 7.1 Hz, 1H), 2.40 (s, 3H).

^{13}C NMR (101 MHz, CDCl$_3$) δ 197.1, 194.7, 165.1, 153.6, 138.7, 137.6, 136.1, 134.6, 130.8 (q, J_{C-F} = 306.6 Hz), 128.9, 128.7, 128.1, 127.2, 126.0, 125.6, 122.6, 42.7, 40.2, 30.6 (q, J_{C-F} = 2.1 Hz), 21.5.

^{19}F NMR (376 MHz, CDCl$_3$) δ -41.3.

HRMS (ESI-TOF) m/z: Calcd for $[M+H]^+$ $C_{20}H_{17}F_3NO_2S_2$ 424.0647; Found: 424.0646.

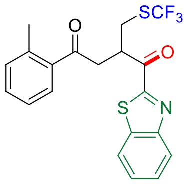

1-(benzo[*d*]thiazol-2-yl)-4-(*o*-tolyl)-2-(((trifluoromethyl)thio)methyl)butane-1,4-dione

The reaction solution was processed as general experimental procedure to afford the

corresponding product **3i** as a yellow oil (16.9 mg, 40%; PE/EA = 20/1).

^{1}H NMR (400 MHz, CDCl$_3$) δ 8.21 (dd, J = 7.5, 1.3 Hz, 1H), 8.00 (dd, J = 7.4, 1.3 Hz, 1H), 7.77 (d, J = 7.0 Hz, 1H), 7.62 – 7.53 (m, 2H), 7.42 – 7.38 (m, 1H), 7.33 – 7.19 (m, 2H), 4.83 – 4.63 (m, 1H), 3.80 (dd, J = 18.0, 8.1 Hz, 1H), 3.67 – 3.44 (m, 2H), 3.27 (dd, J = 13.6, 7.3 Hz, 1H), 2.46 (s, 3H).

^{13}C NMR (101 MHz, CDCl$_3$) δ 200.4, 194.6, 165.0, 153.6, 139.0, 137.6, 136.5, 132.3, 132.1, 130.8 (q, $J_{C\text{-}F}$ = 306.6 Hz), 129.1, 128.1, 127.2, 125.9, 122.6, 43.0, 42.5, 30.7 (q, $J_{C\text{-}F}$ = 2.2 Hz), 21.6.

^{19}F NMR (376 MHz, CDCl$_3$) δ -41.3.

HRMS (ESI-TOF) m/z: Calcd for $[M+H]^+$ $C_{20}H_{17}F_3NO_2S_2$ 424.0647; Found: 424.0656.

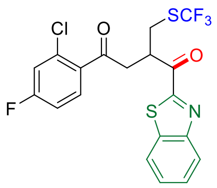

1-(benzo[*d*]thiazol-2-yl)-4-(2-chloro-4-fluorophenyl)-2-(((trifluoromethyl)thio)methyl)butane-1,4-dione

The reaction solution was processed as general experimental procedure to afford the corresponding product **3j** as a yellow oil (25.4 mg, 55%; PE/EA = 20/1).

^{1}H NMR (400 MHz, CDCl$_3$) δ 8.31 – 8.13 (m, 1H), 8.10 – 7.91 (m, 1H), 7.67 (dd, *J* = 8.7, 6.0 Hz, 1H), 7.62 – 7.53 (m, 2H), 7.17 (dd, *J* = 8.4, 2.5 Hz, 1H), 7.07 – 7.03 (m, 1H), 4.80 – 4.73 (m, 1H), 3.82 (dd, *J* = 18.3, 8.3 Hz, 1H), 3.67 – 3.51 (m, 2H), 3.28 (dd, *J* = 13.8, 7.4 Hz, 1H).

^{13}C NMR (101 MHz, CDCl$_3$) δ 198.0, 194.2, 164.7, 164.1 (d, $J_{C\text{-}F}$ = 256.5 Hz), 153.6, 137.6, 133.9 (d, $J_{C\text{-}F}$ = 3.6 Hz), 133.6 (d, $J_{C\text{-}F}$ = 10.7 Hz), 132.0 (d, $J_{C\text{-}F}$ = 9.6 Hz), 130.7 (q, $J_{C\text{-}F}$ = 306.6 Hz), 128.2, 127.3, 125.9, 122.6, 118.5 (d, $J_{C\text{-}F}$ = 24.9 Hz), 114.7 (d, $J_{C\text{-}F}$ = 21.3 Hz), 43.8, 43.2, 30.6 (q, $J_{C\text{-}F}$ = 2.2 Hz).

^{19}F NMR (376 MHz, CDCl$_3$) δ -41.2, -105.4.

HRMS (ESI-TOF) m/z: Calcd for $[M+H]^+$ $C_{19}H_{13}ClF_4NO_2S_2$ 462.0007; Found: 462.0016.

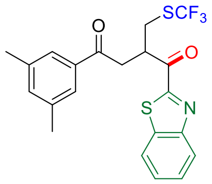

1-(benzo[*d*]thiazol-2-yl)-4-(3,5-dimethylphenyl)-2-(((trifluoromethyl)thio)methyl) butane-1,4-dione

The reaction solution was processed as general experimental procedure to afford the corresponding product **3k** as a yellow oil (17.9 mg, 41%; PE/EA = 20/1).

^{1}H NMR (400 MHz, $CDCl_3$) δ 8.33 – 8.12 (m, 1H), 8.09 – 7.91 (m, 1H), 7.68 – 7.42 (m, 4H), 7.22 (s, 1H), 4.98 – 4.59 (m, 1H), 3.81 (dd, J = 18.0, 7.9 Hz, 1H), 3.63 – 3.51 (m, 2H), 3.28 (dd, J = 13.6, 7.0 Hz, 1H), 2.36 (s, 6H).

^{13}C NMR (101 MHz, $CDCl_3$) δ 197.3, 194.8, 165.1, 153.6, 138.5, 137.6, 136.2, 135.4, 130.8 (q, $J_{C\text{-}F}$ = 306.6 Hz), 128.1, 127.2, 126.1, 126.0, 122.6, 42.7, 40.3, 30.6 (q, $J_{C\text{-}F}$ = 2.2 Hz), 21.4.

^{19}F NMR (376 MHz, $CDCl_3$) δ -41.3.

HRMS (ESI-TOF) m/z: Calcd for $[M+H]^+$ $C_{21}H_{19}F_3NO_2S_2$ 438.0804; Found: 438.0807.

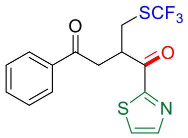

4-phenyl-1-(thiazol-2-yl)-2-(((trifluoromethyl)thio)methyl)butane-1,4-dione

The reaction solution was processed as general experimental procedure to afford the corresponding product **3l** as a yellow oil (23.3 mg, 65%; PE/EA = 20/1).

^{1}H NMR (700 MHz, $CDCl_3$) δ 8.06 (d, J = 3.0 Hz, 1H), 8.01 – 7.90 (m, 2H), 7.73 (d, J = 3.0 Hz, 1H), 7.58 (t, J = 7.4 Hz, 1H), 7.47 (t, J = 7.8 Hz, 2H), 4.73 – 4.55 (m, 1H), 3.80 (dd, J = 17.9, 8.1 Hz, 1H), 3.57 – 3.48 (m, 2H), 3.25 (dd, J = 13.6, 7.2 Hz, 1H).

^{13}C NMR (101 MHz, $CDCl_3$) δ 197.0, 193.1, 165.7, 145.2, 136.2, 133.8, 130.8 (q, $J_{C\text{-}F}$ = 306.6 Hz), 128.8, 128.3, 127.1, 42.6, 39.9, 30.6 (q, $J_{C\text{-}F}$ = 2.2 Hz).

^{19}F NMR (376 MHz, $CDCl_3$) δ -41.3.

HRMS (ESI-TOF) m/z: Calcd for $[M+H]^+$ $C_{15}H_{13}F_3NO_2S_2$ 360.0334; Found: 360.0339.

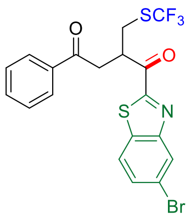

1-(5-bromobenzo[*d*]thiazol-2-yl)-4-phenyl-2-(((trifluoromethyl)thio)methyl)butane-1,4-dione

The reaction solution was processed as general experimental procedure to afford the corresponding product **3m** as a yellow oil (24.8 mg, 51%; PE/EA = 20/1).

^{1}H NMR (400 MHz, CDCl$_3$) δ 8.15 (d, J = 1.8 Hz, 1H), 8.05 (d, J = 8.8 Hz, 1H), 7.99 – 7.92 (m, 2H), 7.69 (dd, J = 8.8, 1.9 Hz, 1H), 7.59 (ddd, J = 8.7, 2.5, 1.2 Hz, 1H), 7.47 (t, J = 7.7 Hz, 2H), 4.75 (dt, J = 13.4, 6.8 Hz, 1H), 3.83 (dd, J = 18.1, 8.4 Hz, 1H), 3.64 (dd, J = 18.1, 5.2 Hz, 1H), 3.53 (dd, J = 13.7, 6.1 Hz, 1H), 3.26 (dd, J = 13.7, 7.3 Hz, 1H).

^{13}C NMR (101 MHz, CDCl$_3$) δ 196.9, 194.5, 165.5, 152.4, 139.1, 136.0, 133.9, 131.0, 130.7 (q, J_{C-F} = 306.6 Hz), 128.9, 128.3, 127.0, 125.1, 122.4, 42.6, 40.2, 30.6 (q, J_{C-F} = 2.2 Hz).

^{19}F NMR (376 MHz, CDCl$_3$) δ -41.2.

HRMS (ESI-TOF) m/z: Calcd for $[M+H]^+$ $C_{19}H_{14}BrF_3NO_2S_2$ 487.9596; Found: 487.9595.

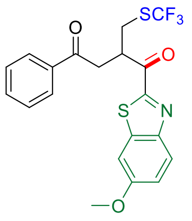

1-(6-methoxybenzo[*d*]thiazol-2-yl)-4-phenyl-2-(((trifluoromethyl)thio)methyl)butane-1,4-dione

The reaction solution was processed as general experimental procedure to afford the corresponding product **3n** as a yellow oil (21.1 mg, 48%; PE/EA = 20/1).

^{1}H NMR (400 MHz, CDCl$_3$) δ 8.17 – 7.89 (m, 3H), 7.74 – 7.52 (m, 1H), 7.52 – 7.40 (m, 2H), 7.38 (d, J = 2.5 Hz, 1H), 7.18 (dd, J = 9.1, 2.5 Hz, 1H), 4.86 – 4.67 (m, 1H), 3.92 (s, 3H), 3.88 – 3.77 (m, 1H), 3.64 – 3.47 (m, 2H), 3.28 (dd, J = 13.6, 7.2 Hz, 1H).

^{13}C NMR (101 MHz, CDCl$_3$) δ 197.0, 194.3, 162.4, 160.1, 148.2, 139.8, 136.2, 133.7, 130.8 (q, $J_{C\text{-}F}$ = 306.6 Hz), 128.8, 128.3, 126.7, 118.0, 103.6, 56.0, 42.5, 40.0, 30.7 (q, $J_{C\text{-}F}$ = 2.1 Hz).

^{19}F NMR (376 MHz, CDCl$_3$) δ -41.3.

HRMS (ESI-TOF) m/z: Calcd for $[M+H]^+$ $C_{20}H_{17}F_3NO_3S_2$ 440.0596; Found: 440.0599.

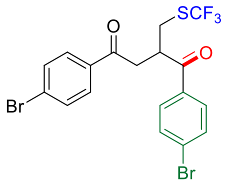

1,4-bis(4-bromophenyl)-2-(((trifluoromethyl)thio)methyl)butane-1,4-dione

The reaction solution was processed as general experimental procedure to afford the corresponding product **3o** as a yellow oil (23.4 mg, 46%; PE/EA = 20/1).

^{1}H NMR (400 MHz, CDCl$_3$) δ 7.94 – 7.85 (m, 2H), 7.84 – 7.77 (m, 2H), 7.66 (d, J = 8.6 Hz, 2H), 7.63 – 7.56 (m, 2H), 4.49 – 4.27 (m, 1H), 3.60 (dd, J = 18.1, 8.2 Hz, 1H), 3.40 (dd, J = 18.1, 5.0 Hz, 1H), 3.27 (dd, J = 13.9, 6.1 Hz, 1H), 3.03 (dd, J = 13.9, 7.7 Hz, 1H).

^{13}C NMR (101 MHz, CDCl$_3$) δ 199.6, 196.1, 134.8, 134.6, 132.4, 132.2, 130.8 (q, $J_{C\text{-}F}$ = 306.7 Hz), 130.2, 129.7, 129.3, 129.2, 41.3, 40.4, 31.2 (q, $J_{C\text{-}F}$ = 2.1 Hz).

^{19}F NMR (376 MHz, CDCl$_3$) δ -41.2.

HRMS (ESI-TOF) m/z: Calcd for $[M+H]^+$ $C_{18}H_{14}Br_2F_3O_2S$ 508.9028; Found: 508.9021.

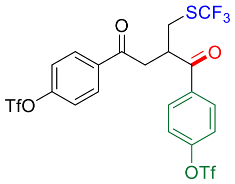

(2-(((trifluoromethyl)thio)methyl)succinyl)bis(4,1-phenylene) bis(trifluoromethanesulfonate)

The reaction solution was processed as general experimental procedure to afford the corresponding product **3p** as a yellow oil (27.2 mg, 42%; PE/EA = 20/1).

^{1}H NMR (400 MHz, CDCl$_3$) δ 8.15 (d, J = 8.8 Hz, 2H), 8.05 (d, J = 8.8 Hz, 2H), 7.45 (d, J = 8.7 Hz, 2H), 7.39 (d, J = 8.8 Hz, 2H), 4.60 – 4.20 (m, 1H), 3.69 (dd, J = 18.2, 8.7 Hz, 1H), 3.47 (dd, J = 18.1, 4.5 Hz, 1H), 3.30 (dd, J = 14.1, 5.9 Hz, 1H), 3.03 (dd, J = 14.1, 8.0 Hz, 1H).

^{13}C NMR (101 MHz, CDCl$_3$) δ 198.8, 195.4, 153.0 (d, J_{C-F} = 5.6 Hz), 135.7 (d, J_{C-F} = 3.0 Hz), 131.1, 130.7 (q, J_{C-F} = 307.0 Hz), 130.6, 127.4 (d, J_{C-F} = 24.7 Hz), 122.1 (d, J_{C-F} = 15.7 Hz), 118.8 (q, J_{C-F} = 320.8 Hz), 118.8 (q, J_{C-F} = 320.7 Hz), 41.55, 40.71, 31.1 (q, J_{C-F} = 2.0 Hz).

^{19}F NMR (376 MHz, CDCl$_3$) δ -41.1, -72.7, -72.7.

HRMS (ESI-TOF) m/z: Calcd for $[M+H]^+$ $C_{20}H_{14}F_9O_8S_3^+$ 648.9702; Found: 648.9711.

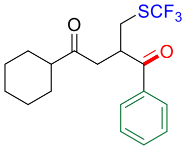

4-cyclohexyl-1-phenyl-2-(((trifluoromethyl)thio)methyl)butane-1,4-dione

The reaction solution was processed as general experimental procedure to afford the corresponding product **3q** as a yellow oil (11.8 mg, 33%; PE/EA = 20/1).

^{1}H NMR (400 MHz, CDCl$_3$) δ 8.12 – 7.78 (m, 2H), 7.60 (t, J = 7.4 Hz, 1H), 7.49 (t, J = 7.6 Hz, 2H), 4.35 – 4.21 (m, 1H), 3.21 (dd, J = 13.7, 6.2 Hz, 1H), 3.08 (dd, J = 18.2, 7.4 Hz, 1H), 2.98 – 2.83 (m, 2H), 2.45 – 2.27 (m, 1H), 1.94 – 1.61 (m, 5H), 1.33 – 1.18 (m, 5H).

^{13}C NMR (101 MHz, CDCl$_3$) δ 211.1, 200.7, 135.7, 133.8, 130.8 (q, $J_{C\text{-}F}$ = 306.5 Hz), 129.0, 128.7, 50.8, 42.0, 41.1, 31.0 (q, $J_{C\text{-}F}$ = 2.1 Hz), 28.5, 28.5, 25.9, 25.7.

^{19}F NMR (376 MHz, CDCl$_3$) δ -41.4.

HRMS (ESI-TOF) m/z: Calcd for $[M+H]^+$ $C_{18}H_{22}F_3O_2S$ 359.1287; Found: 359.1297.

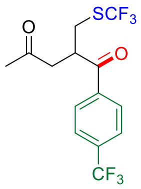

1-(4-(trifluoromethyl)phenyl)-2-(((trifluoromethyl)thio)methyl)pentane-1,4-dione

The reaction solution was processed as general experimental procedure to afford the corresponding product **3r** as a yellow oil (14.3 mg, 40%; PE/EA = 20/1).

^{1}H NMR (400 MHz, CDCl$_3$) δ 8.08 (d, J = 8.1 Hz, 2H), 7.77 (d, J = 8.2 Hz, 2H), 4.39 – 4.07 (m, 1H), 3.22 – 3.07 (m, 2H), 3.01 – 2.83 (m, 2H), 2.18 (s, 3H).

^{13}C NMR (101 MHz, CDCl$_3$) δ 205.4, 199.9, 138.7, 135.0 (q, $J_{C\text{-}F}$ = 32.8 Hz), 130.8 (q, $J_{C\text{-}F}$ = 306.5 Hz), 126.1 (q, $J_{C\text{-}F}$ = 3.7 Hz), 123.6 (q, $J_{C\text{-}F}$ = 272.8 Hz), 45.0, 41.5, 30.8 (q, $J_{C\text{-}F}$ = 2.2 Hz), 29.8.

^{19}F NMR (376 MHz, CDCl$_3$) δ -41.3, -63.2.

HRMS (ESI-TOF) m/z: Calcd for $[M+H]^+$ $C_{14}H_{13}F_6O_2S$ 359.0535; Found: 359.0535.

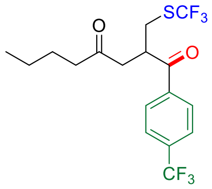

1-(4-(trifluoromethyl)phenyl)-2-(((trifluoromethyl)thio)methyl)octane-1,4-dione

The reaction solution was processed as general experimental procedure to afford the corresponding product **3s** as a yellow oil (17.6 mg, 44%; PE/EA = 20/1).

^{1}H NMR (400 MHz, CDCl$_3$) δ 8.08 (d, J = 8.1 Hz, 2H), 7.76 (d, J = 8.2 Hz, 2H), 4.60 – 3.82 (m, 1H), 3.21 – 2.85 (m, 4H), 2.43 (t, J = 7.4 Hz, 2H), 1.69 – 1.42 (m, 2H), 1.33 – 1.24 (m, 2H), 1.02 – 0.72 (m, 3H).

^{13}C NMR (101 MHz, CDCl$_3$) δ 208.1, 200.1, 138.74, 135.0 (q, $J_{C\text{-}F}$ = 32.8 Hz), 130.8 (q, $J_{C\text{-}F}$ = 306.6 Hz), 129.0, 126.1 (q, $J_{C\text{-}F}$ = 3.7 Hz), 123.7 (q, $J_{C\text{-}F}$ = 272.7 Hz), 44.3, 42.5, 41.5, 30.9 (q, $J_{C\text{-}F}$ = 2.1 Hz), 25.9, 22.4, 13.9.

^{19}F NMR (376 MHz, CDCl$_3$) δ -41.3, -63.2.

HRMS (ESI-TOF) m/z: Calcd for $[M+H]^+$ $C_{17}H_{19}F_6O_2S$ 401.0004; Found: 401.0004.

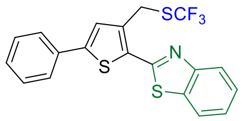

2-(5-phenyl-3-(((trifluoromethyl)thio)methyl)thiophen-2-yl)benzo[d]thiazole

The reaction solution was processed as general experimental procedure to afford the corresponding product **4a** as a yellow oil (45.6 mg, 56%; PE/EA = 20/1).

^{1}H NMR (700 MHz, CDCl$_3$) δ 8.05 (d, J = 8.1 Hz, 1H), 7.89 (d, J = 7.9 Hz, 1H), 7.65 (d, J = 7.2 Hz, 2H), 7.56 – 7.46 (m, 1H), 7.48 – 7.33 (m, 5H), 4.66 (s, 2H).

^{13}C NMR (176 MHz, CDCl$_3$) δ 159.8, 153.8, 146.7, 138.0, 134.7, 133.0, 132.2, 131.3 (q, $J_{C\text{-}F}$ = 307.3 Hz), 129.3, 128.9, 126.8, 126.7, 126.1, 125.6, 123.4, 121.6, 28.6 (q, $J_{C\text{-}F}$ = 2.4 Hz).

^{19}F NMR (376 MHz, CDCl$_3$) δ -41.4.

HRMS (ESI-TOF) m/z: Calcd for $[M+H]^+$ $C_{19}H_{13}F_3NS_3^+$ 408.0157; Found: 408.0161.

6. The NMR Spectrum

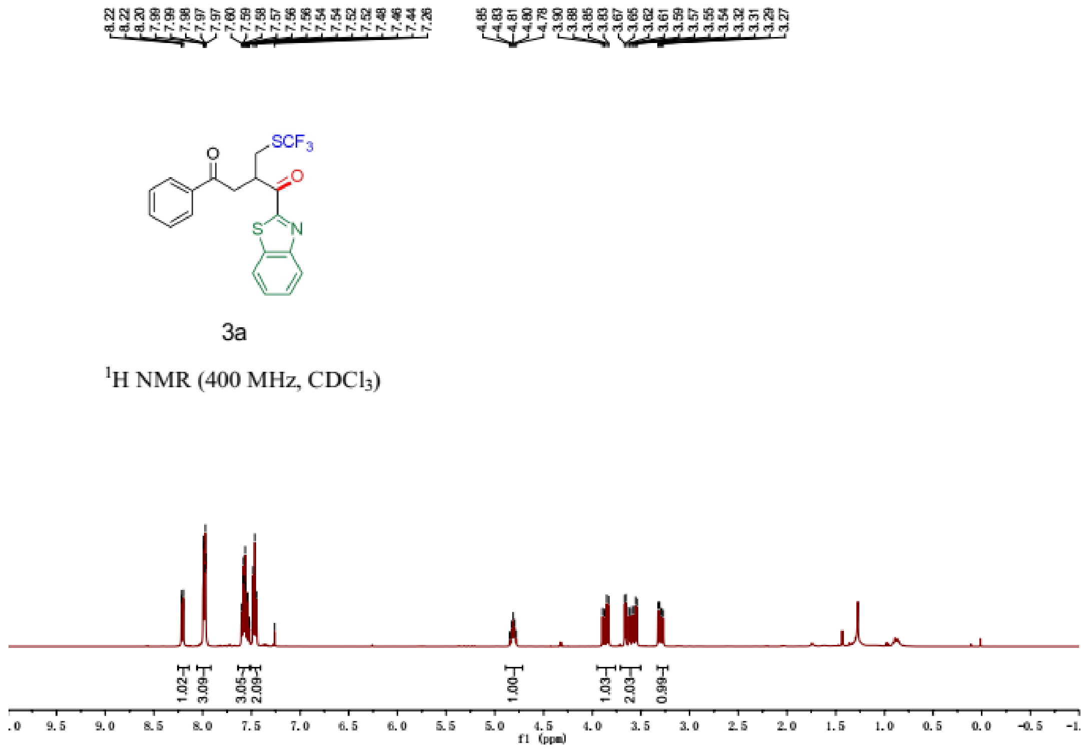

3a

^{1}H NMR (400 MHz, $CDCl_3$)

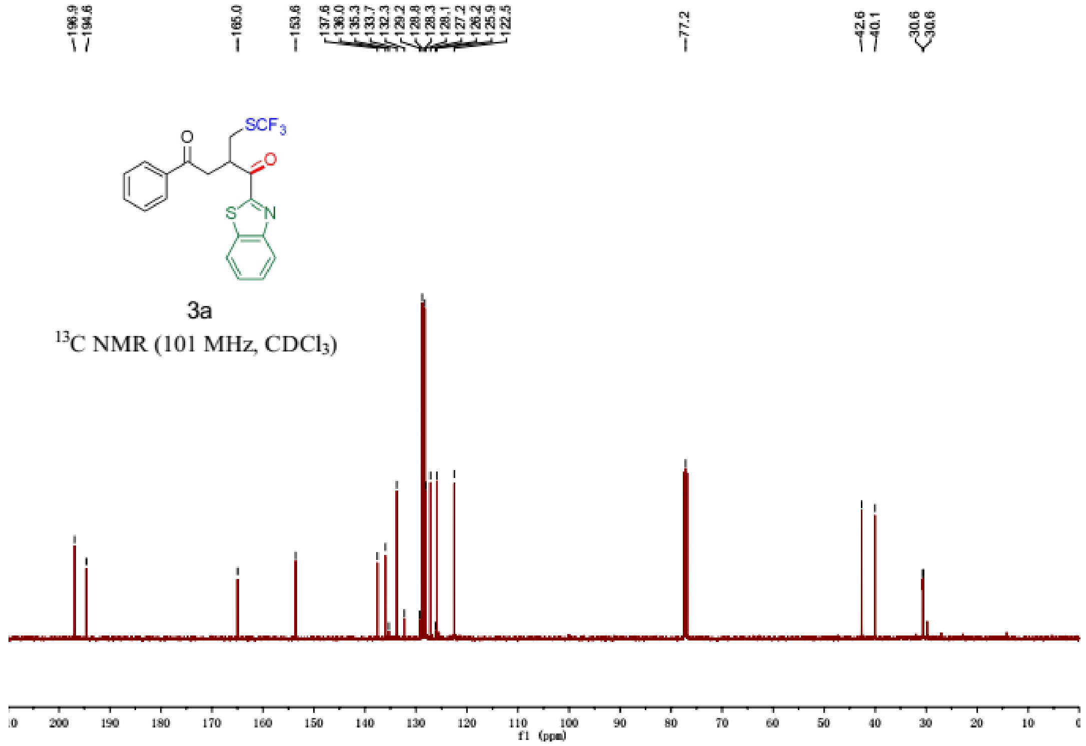

3a

^{13}C NMR (101 MHz, $CDCl_3$)

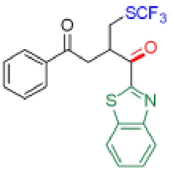

3a

^{19}F NMR (376 MHz, $CDCl_3$)

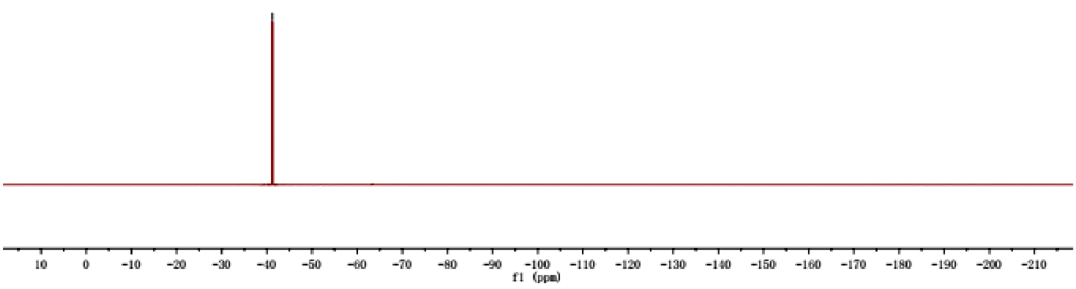

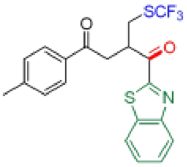

3b

^{1}H NMR (400 MHz, $CDCl_3$)

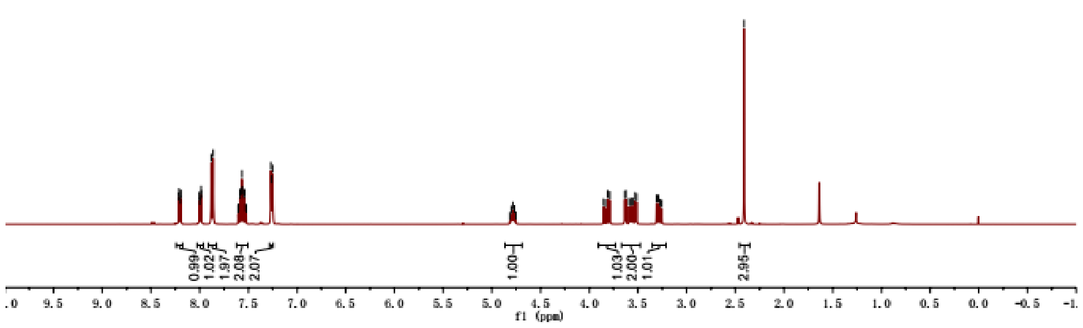

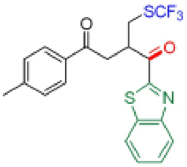

3b

^{13}C NMR (101 MHz, $CDCl_3$)

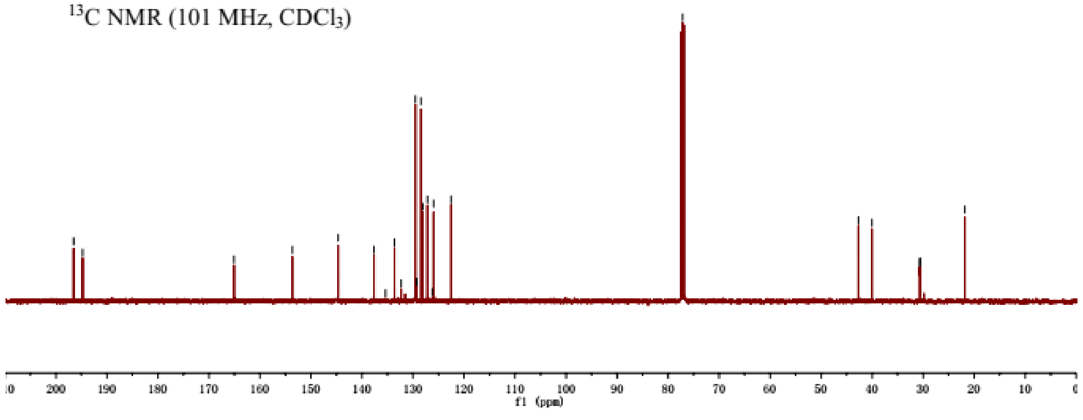

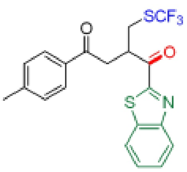

3b

^{19}F NMR (376 MHz, $CDCl_3$)

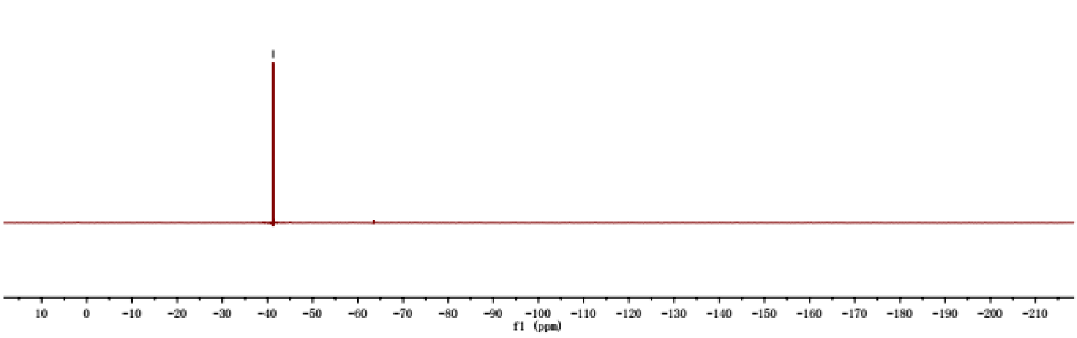

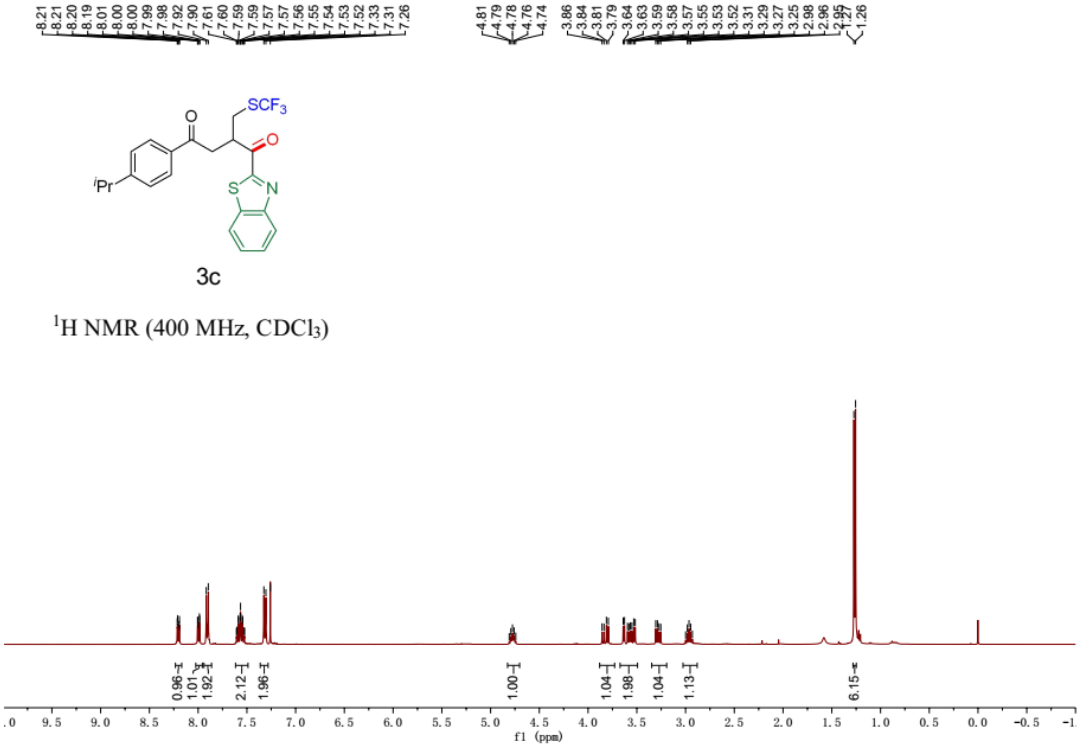

3c

^{1}H NMR (400 MHz, $CDCl_3$)

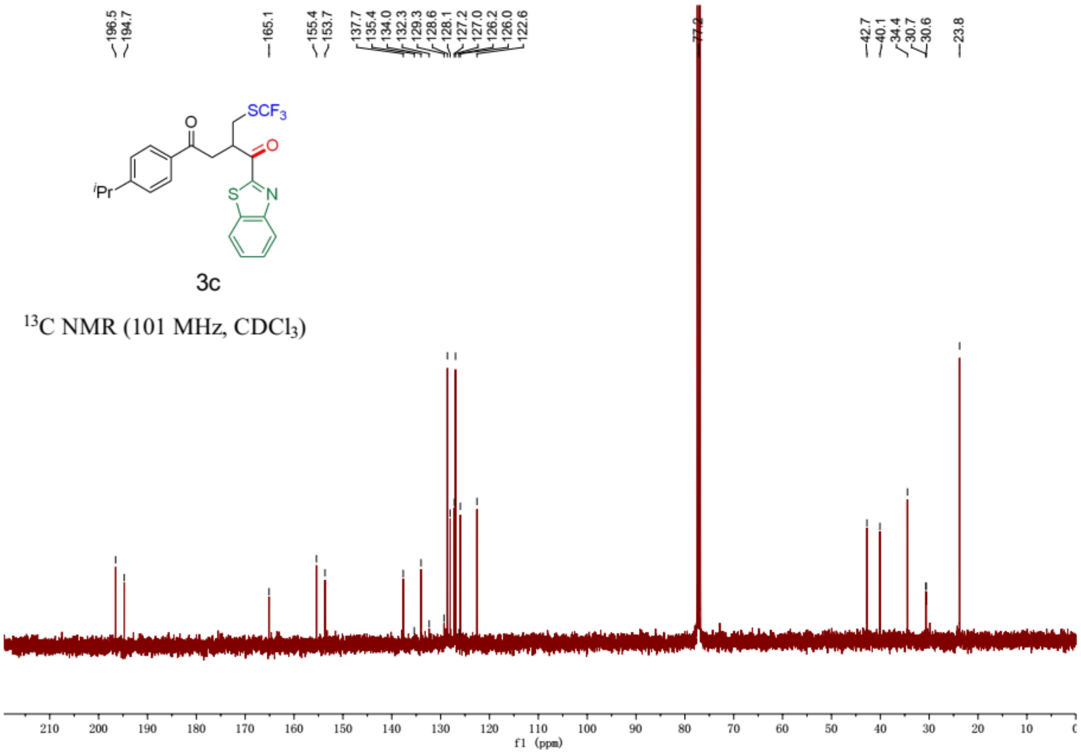

3c

^{13}C NMR (101 MHz, $CDCl_3$)

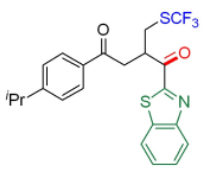

3c

^{19}F NMR (376 MHz, $CDCl_3$)

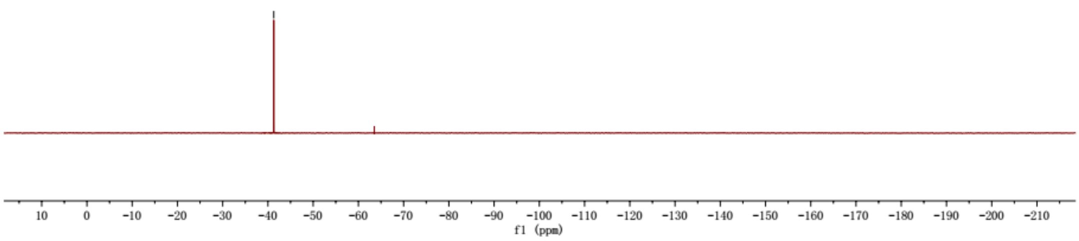

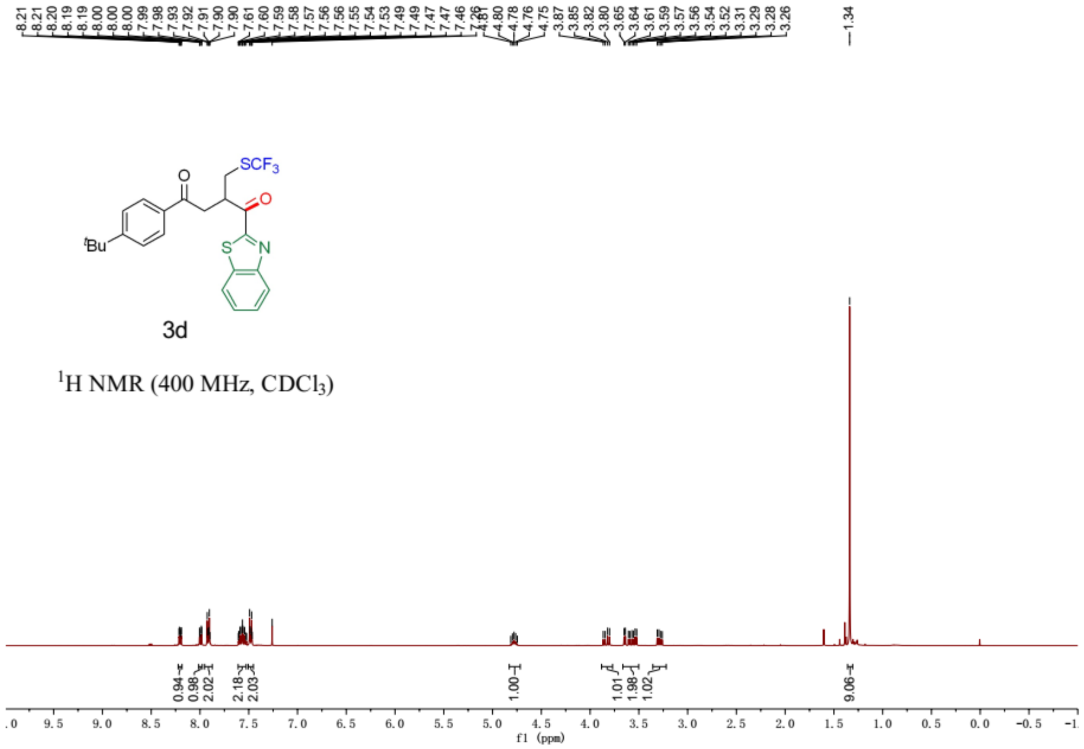
SCF3
O
O
S
N
tBu
3d
1H NMR (400 MHz, CDCl3)
f1 (ppm)

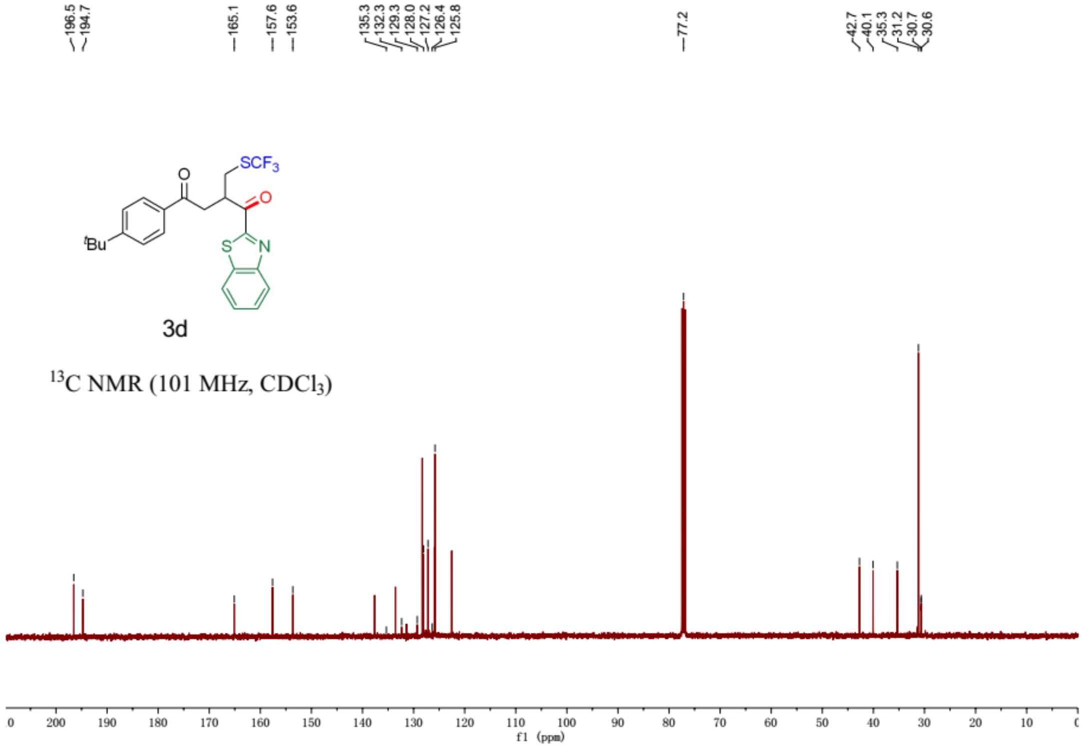
196.5
194.7
165.1
157.6
153.6
135.3
132.3
129.3
128.0
127.2
126.4
125.8
77.2
42.7
40.1
35.3
31.2
30.7
30.6
SCF3
O
O
S
N
tBu
3d
13C NMR (101 MHz, CDCl3)
f1 (ppm)

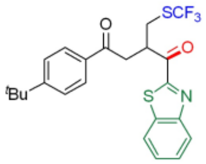

3d

^{19}F NMR (376 MHz, $CDCl_3$)

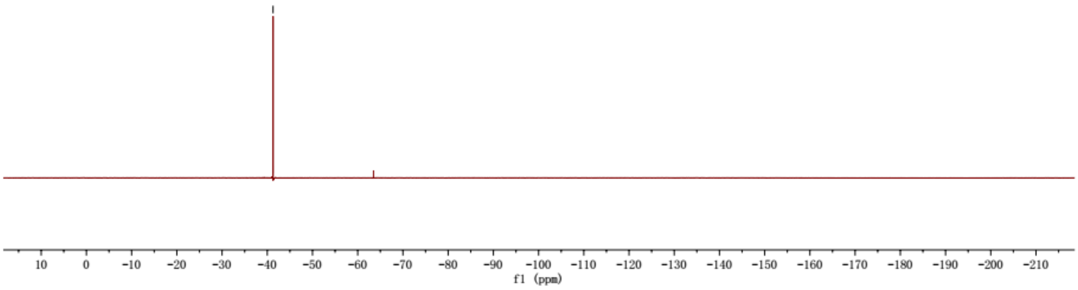

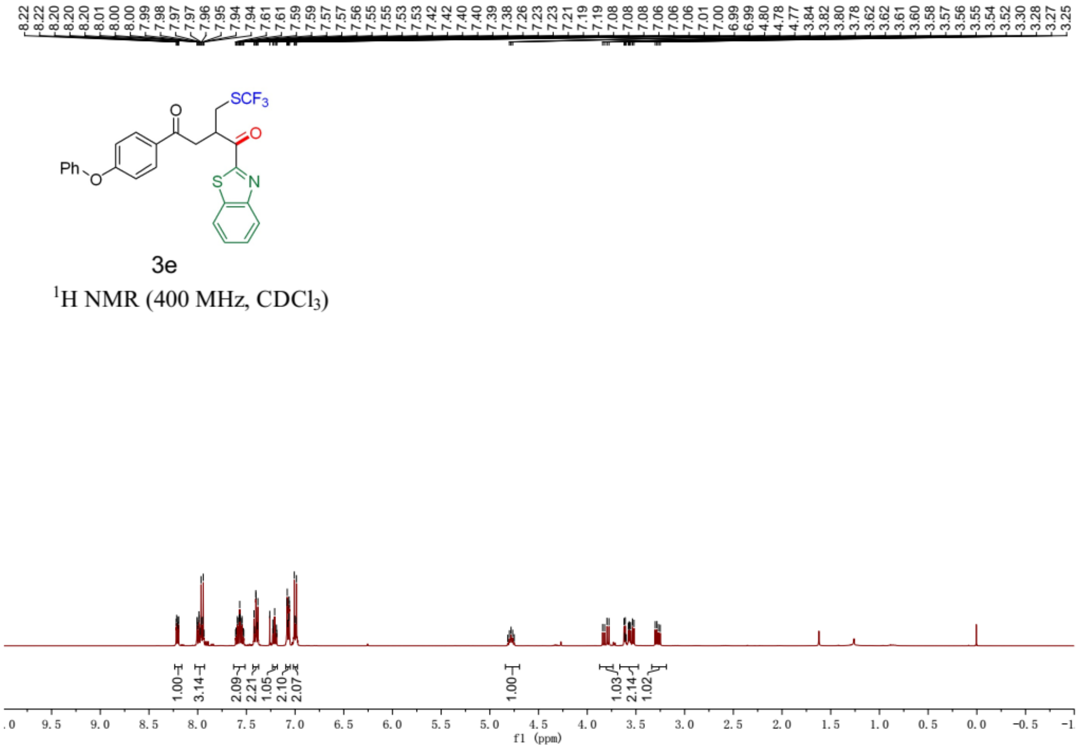

3e

^{1}H NMR (400 MHz, CDCl$_3$)

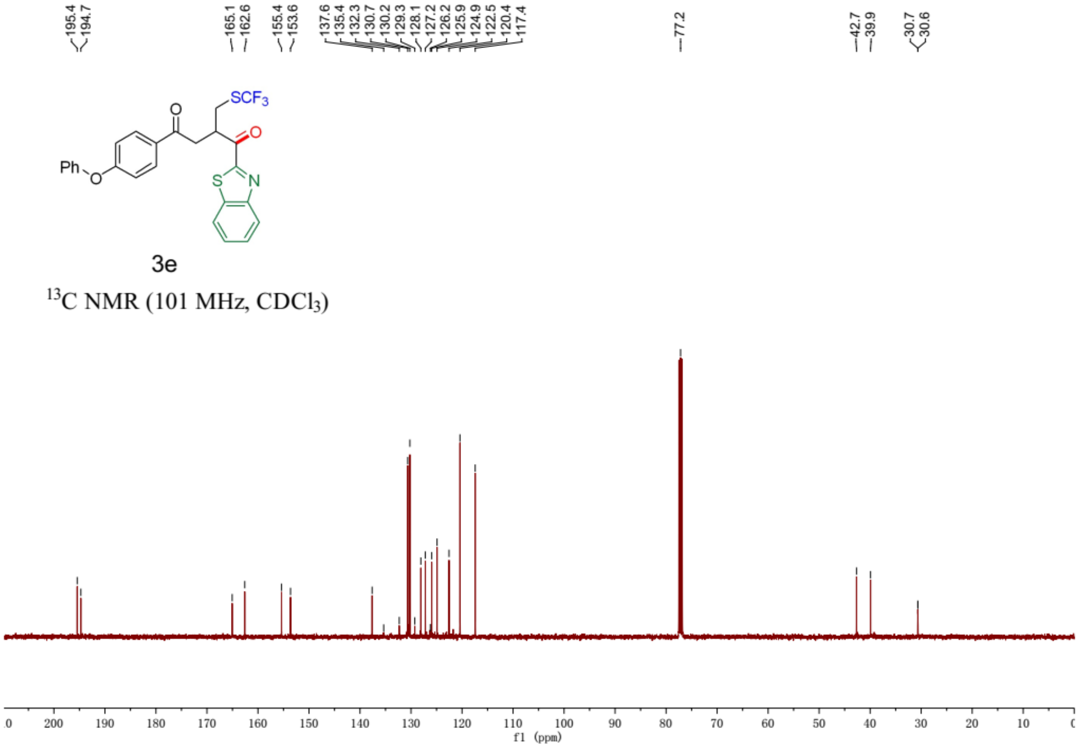

3e

^{13}C NMR (101 MHz, CDCl$_3$)

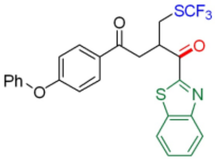

3e

^{19}F NMR (376 MHz, $CDCl_3$)

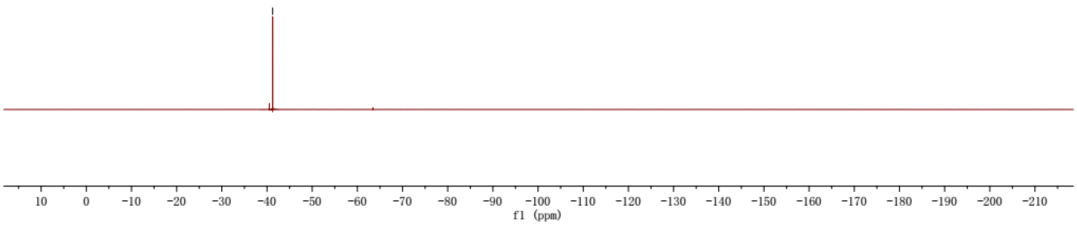

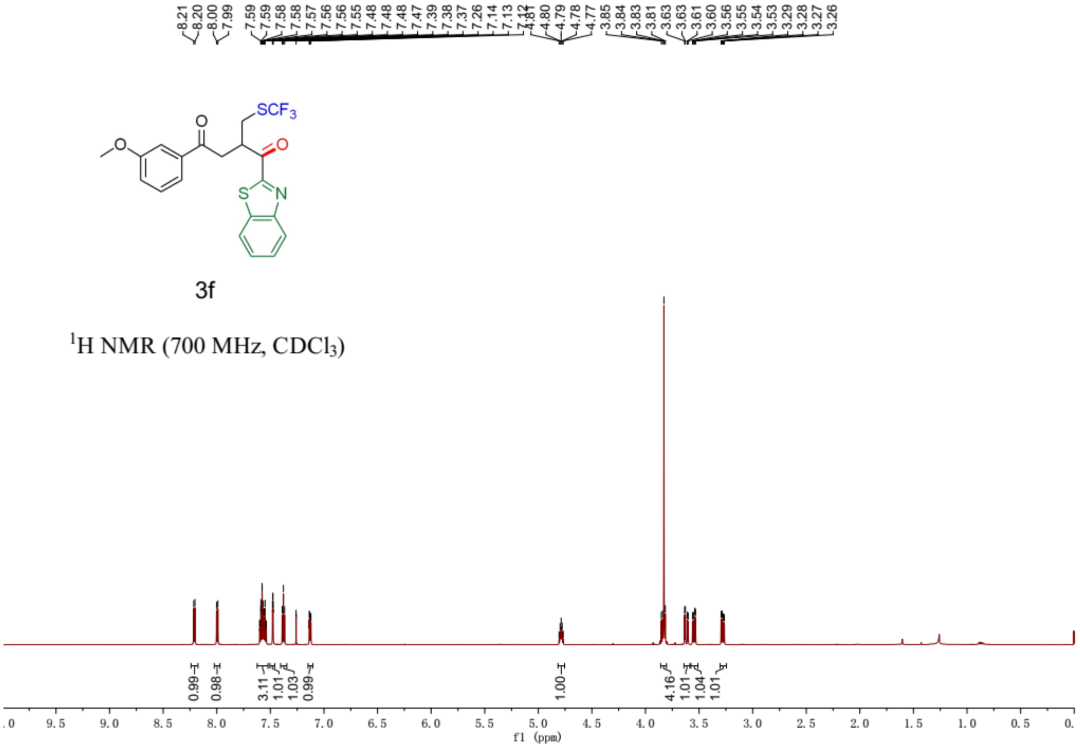
SCF3
O
O
S
N
3f
1H NMR (700 MHz, CDCl3)
f1 (ppm)

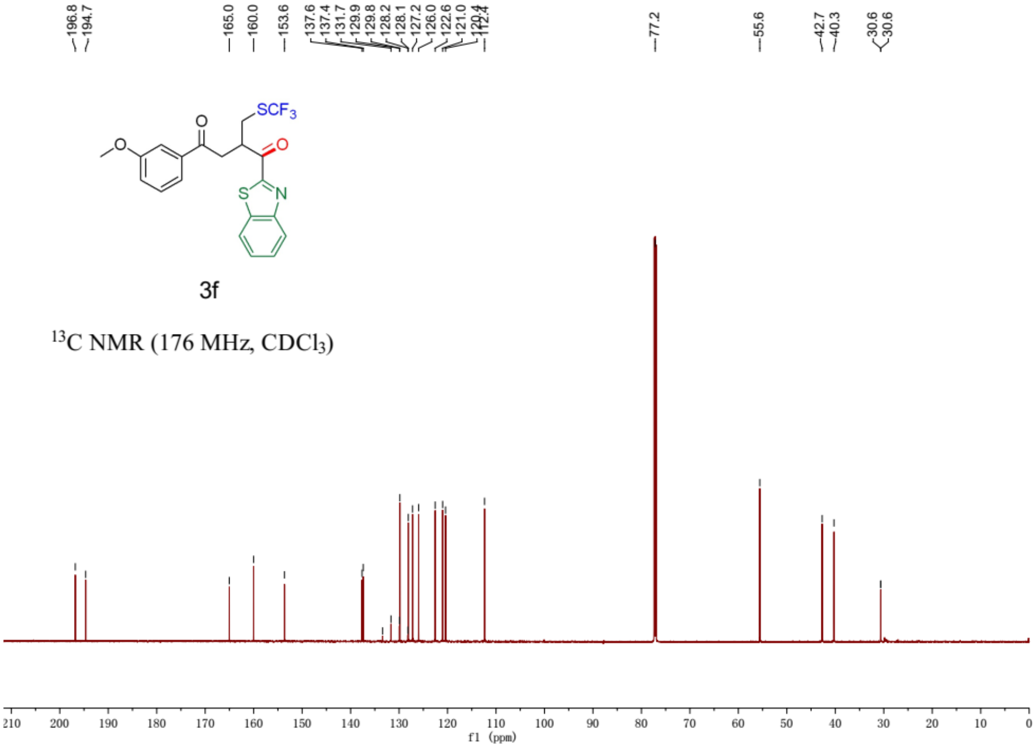
SCF3
O
O
S
N
3f
13C NMR (176 MHz, CDCl3)
f1 (ppm)

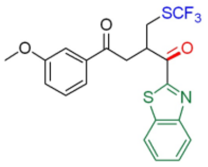

3f

^{19}F NMR (376 MHz, $CDCl_3$)

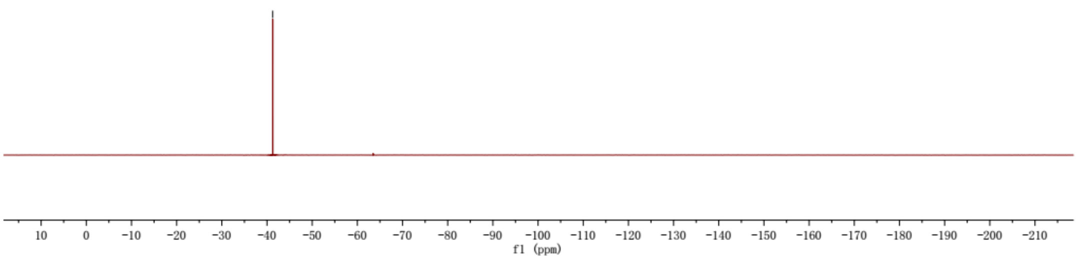

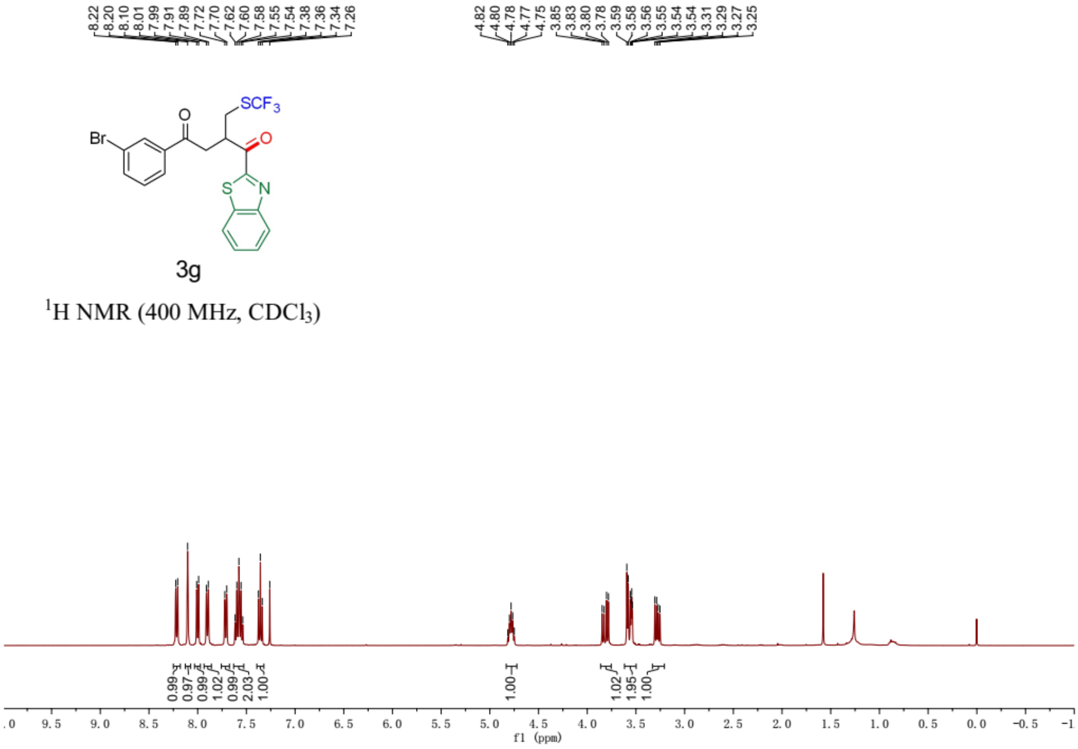

3g

^{1}H NMR (400 MHz, $CDCl_3$)

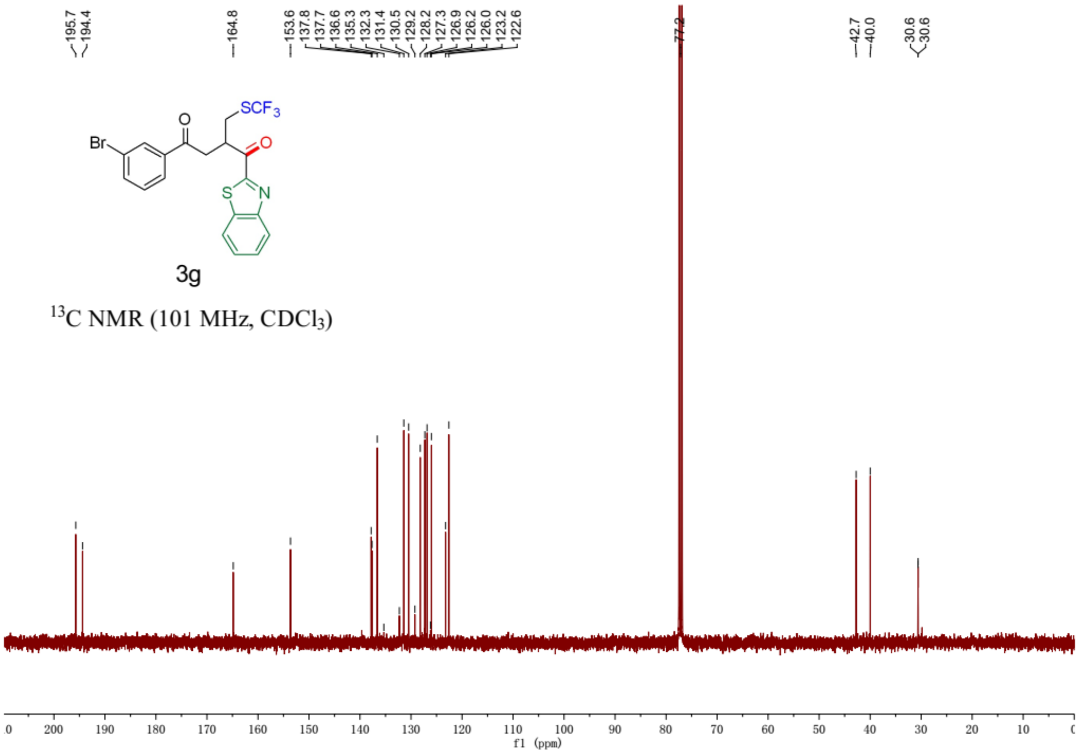

3g

^{13}C NMR (101 MHz, $CDCl_3$)

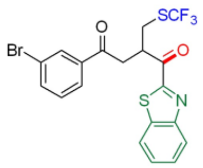

3g

^{19}F NMR (376 MHz, $CDCl_3$)

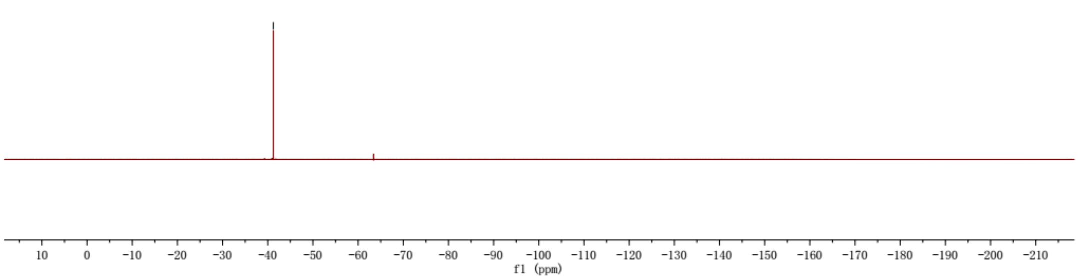

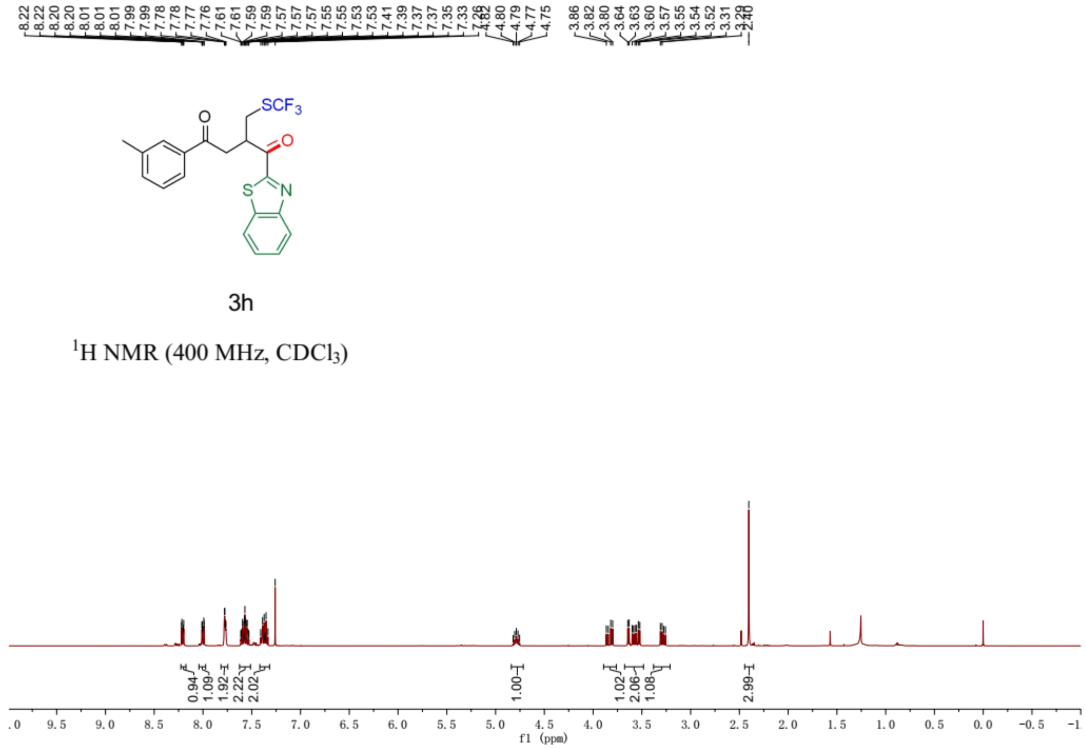

3h

^{1}H NMR (400 MHz, $CDCl_3$)

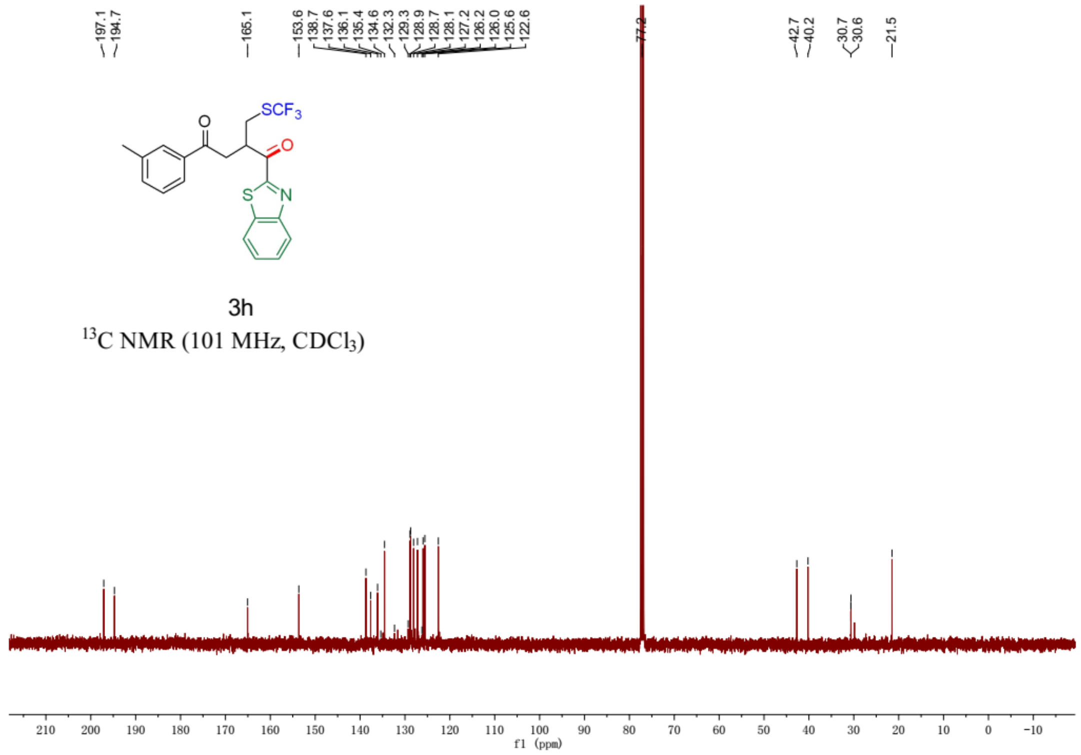

3h

^{13}C NMR (101 MHz, $CDCl_3$)

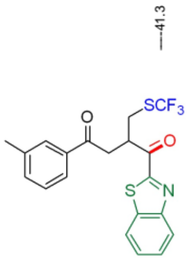

3h

^{19}F NMR (376 MHz, $CDCl_3$)

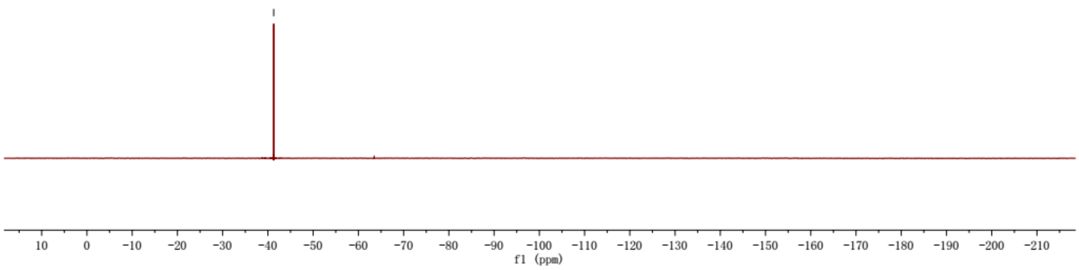

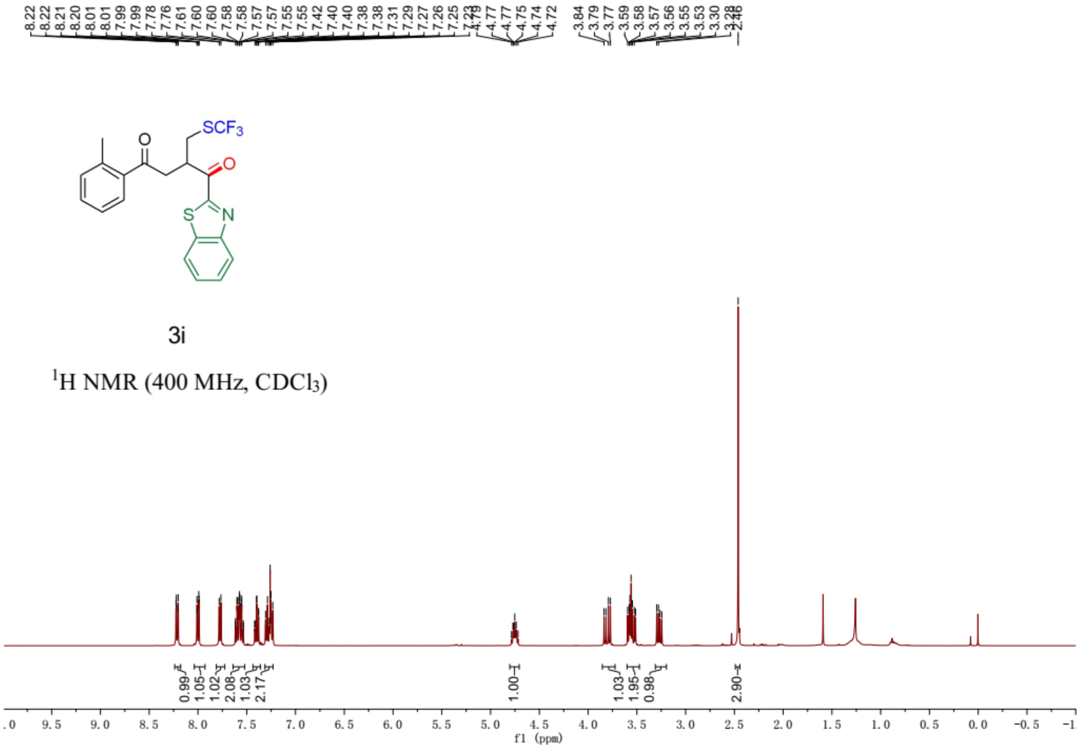

3i

^{1}H NMR (400 MHz, $CDCl_3$)

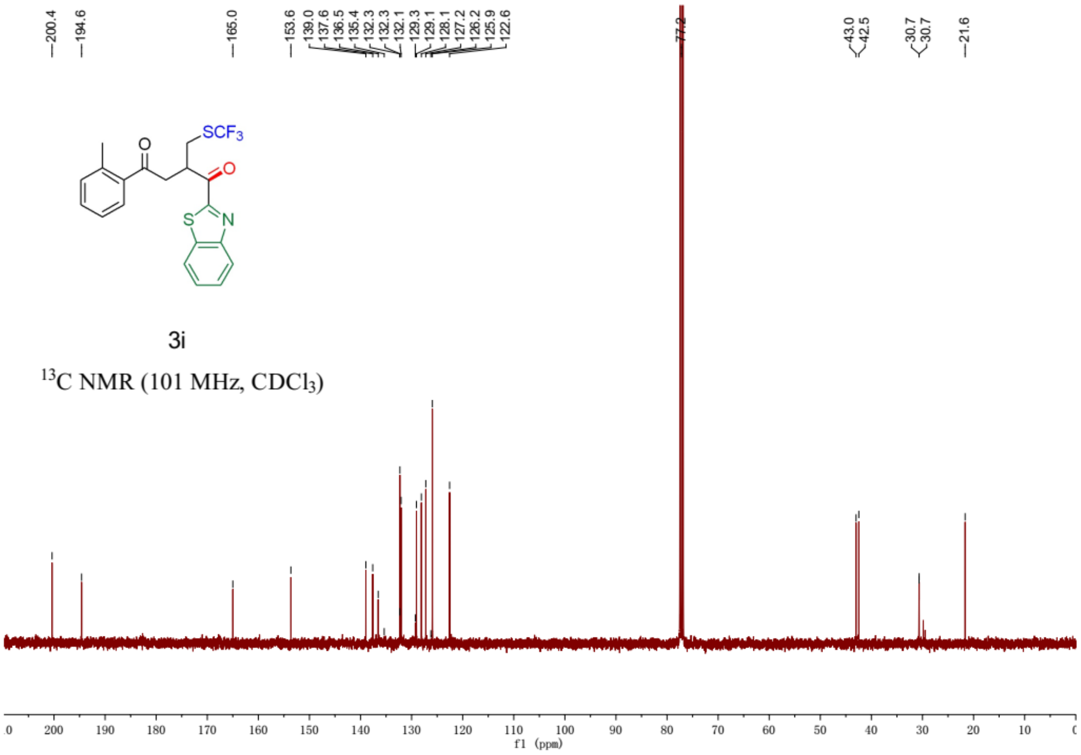

3i

^{13}C NMR (101 MHz, $CDCl_3$)

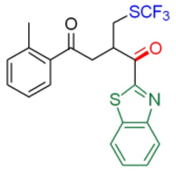

3i

^{19}F NMR (376 MHz, $CDCl_3$)

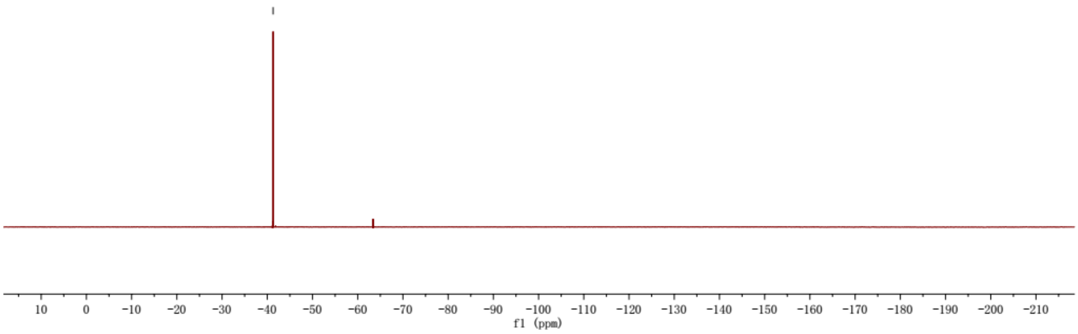

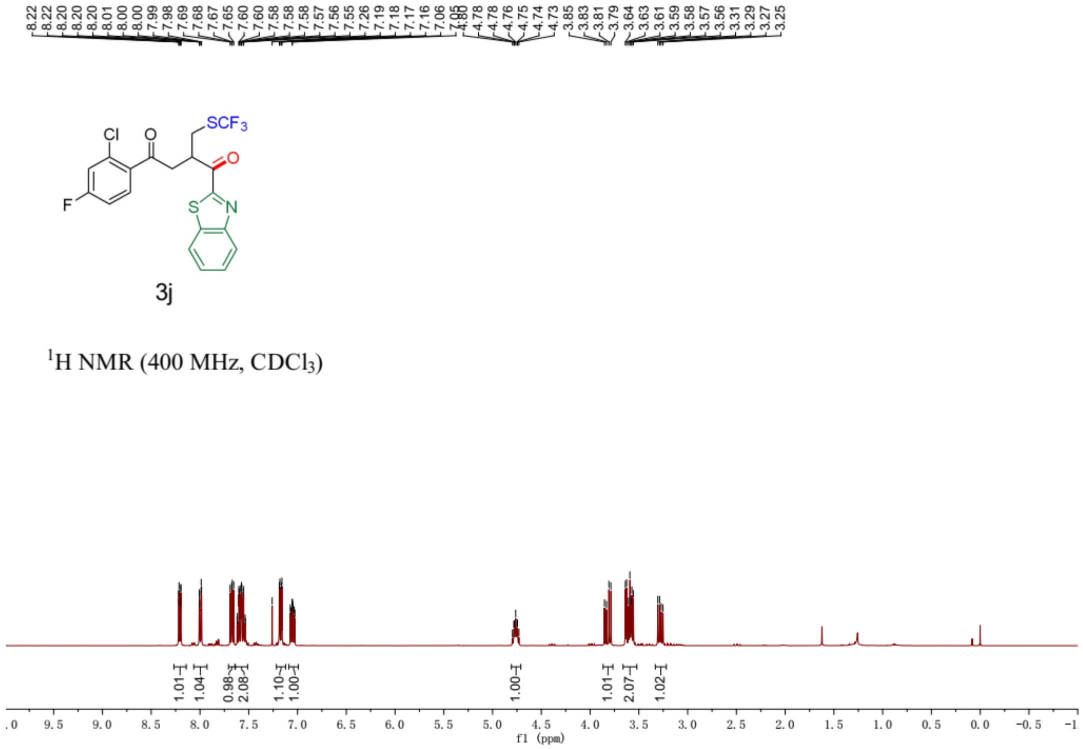
3j
^{1}H NMR (400 MHz, $CDCl_3$)
f1 (ppm)

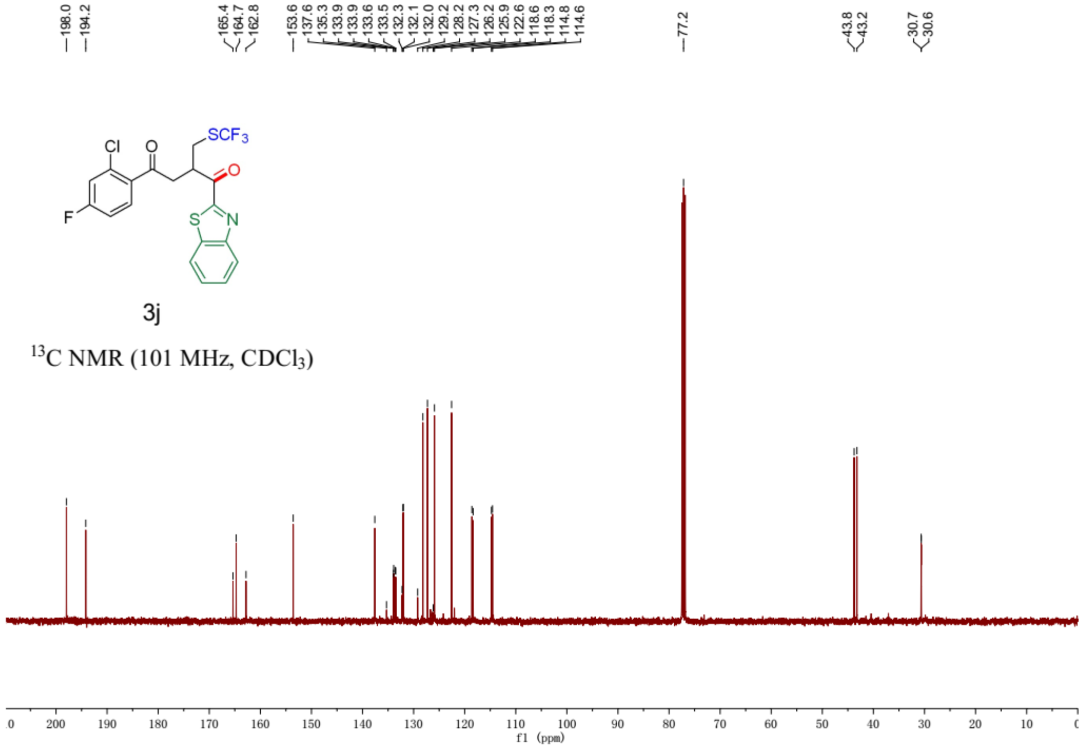
3j
^{13}C NMR (101 MHz, $CDCl_3$)
f1 (ppm)

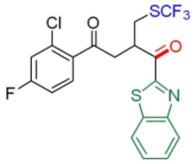

3j

^{19}F NMR (376 MHz, $CDCl_3$)

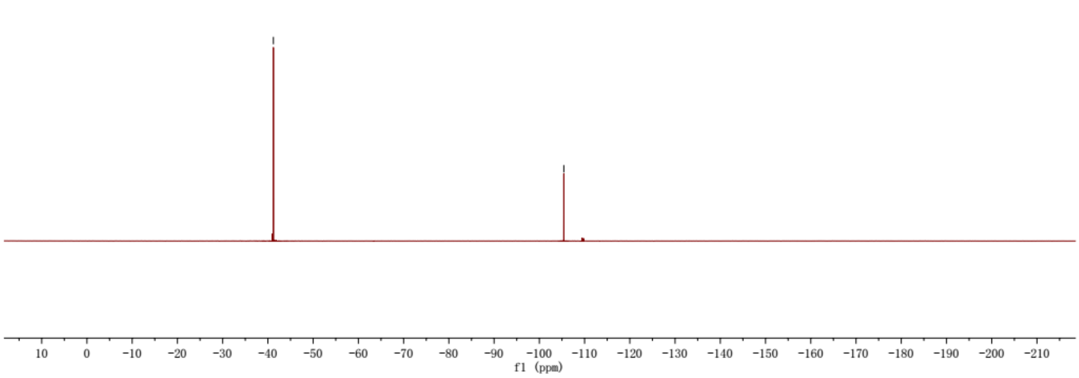

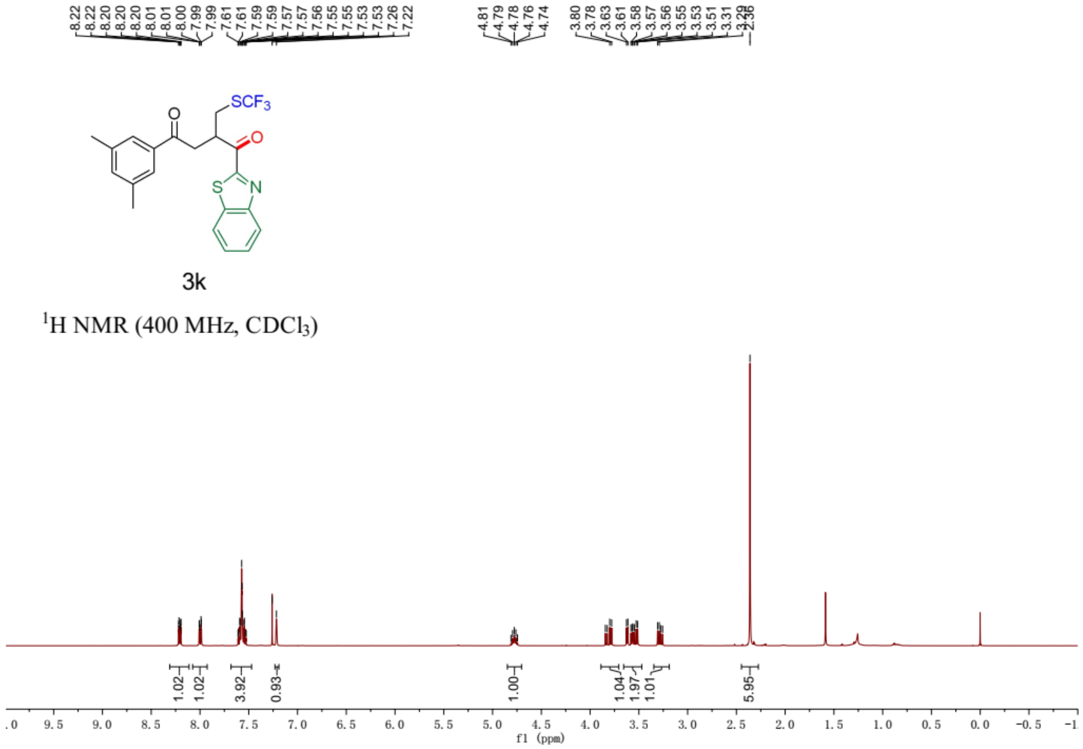

3k

^{1}H NMR (400 MHz, $CDCl_3$)

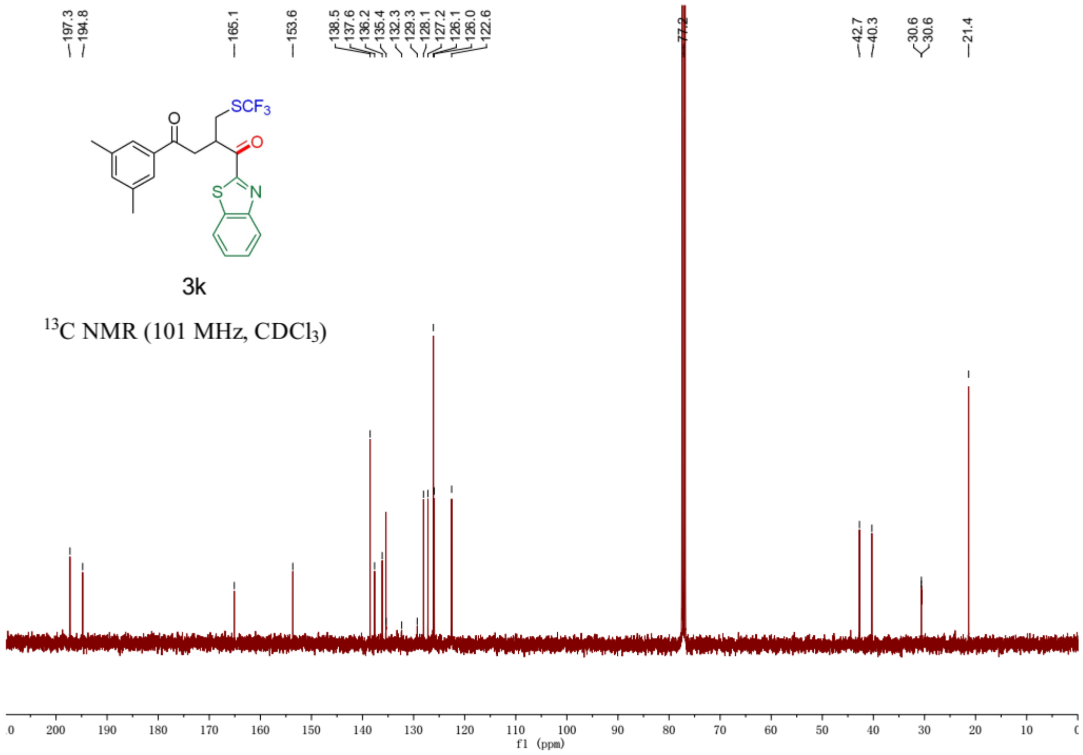

3k

^{13}C NMR (101 MHz, $CDCl_3$)

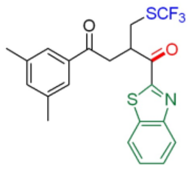

3k

^{19}F NMR (376 MHz, $CDCl_3$)

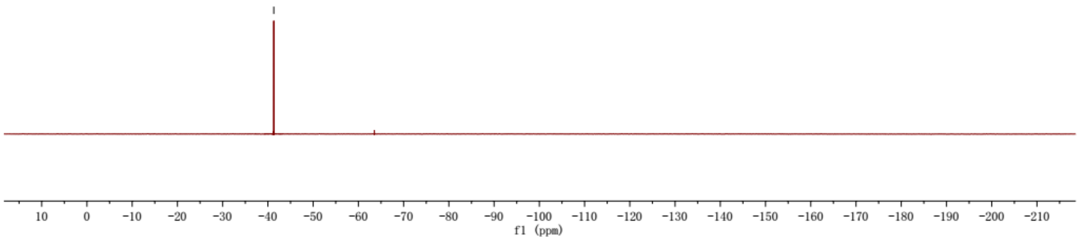

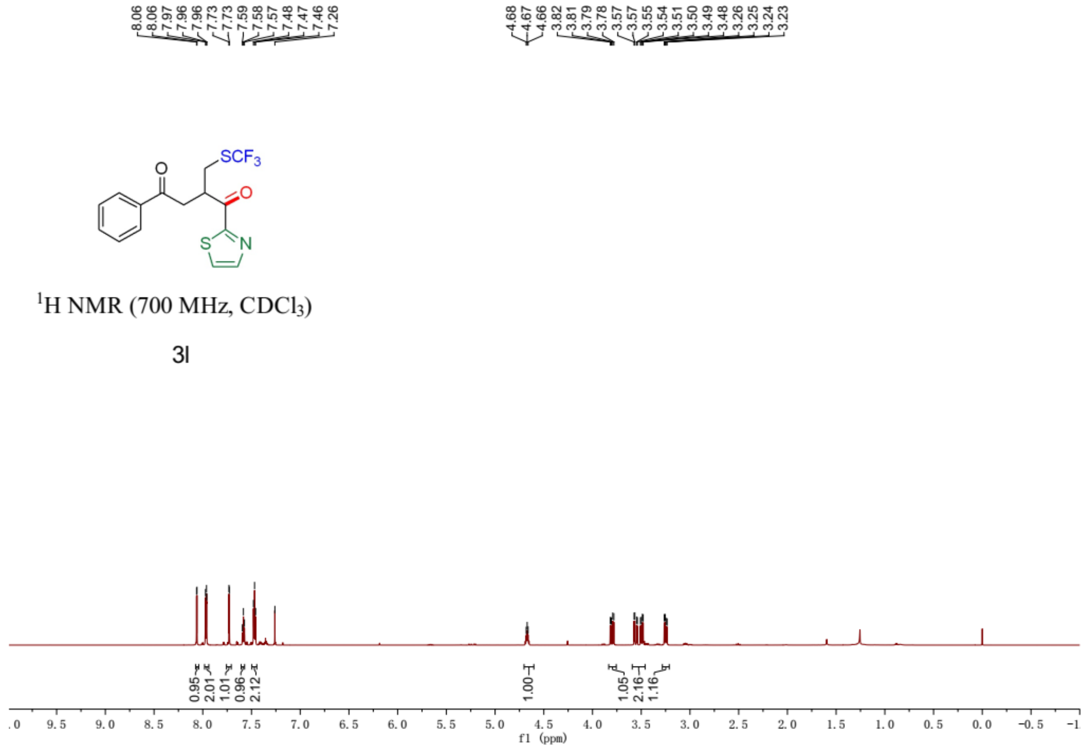
8.06
8.06
7.97
7.96
7.96
7.73
7.73
7.59
7.58
7.57
7.48
7.47
7.46
7.26
4.68
4.67
4.66
3.82
3.81
3.79
3.78
3.57
3.57
3.55
3.54
3.51
3.50
3.49
3.48
3.26
3.25
3.24
3.23
SCF3
O
O
S
N
1H NMR (700 MHz, CDCl3)
3l
0.95
2.01
1.01
0.96
2.12
1.00
1.05
2.16
1.16
f1 (ppm)

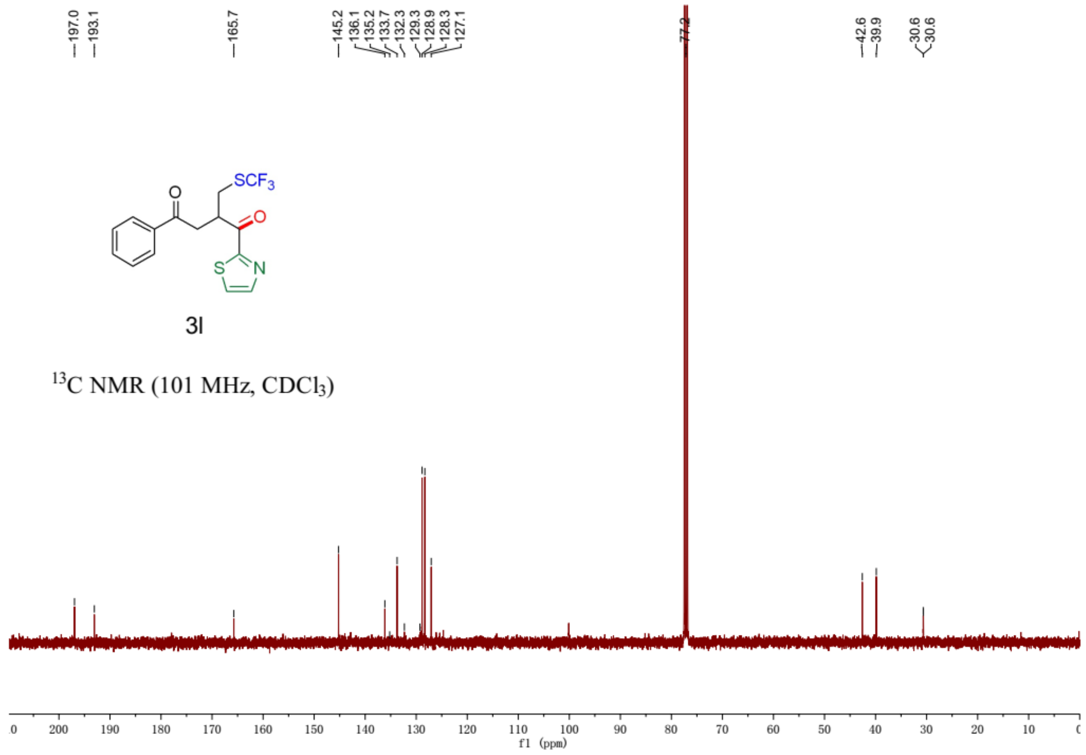
197.0
193.1
165.7
145.2
136.1
135.2
133.7
132.3
129.3
128.9
128.3
127.1
77.2
42.6
39.9
30.6
30.6
SCF3
O
O
S
N
3l
13C NMR (101 MHz, CDCl3)
f1 (ppm)

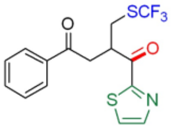

3l

^{19}F NMR (376 MHz, $CDCl_3$)

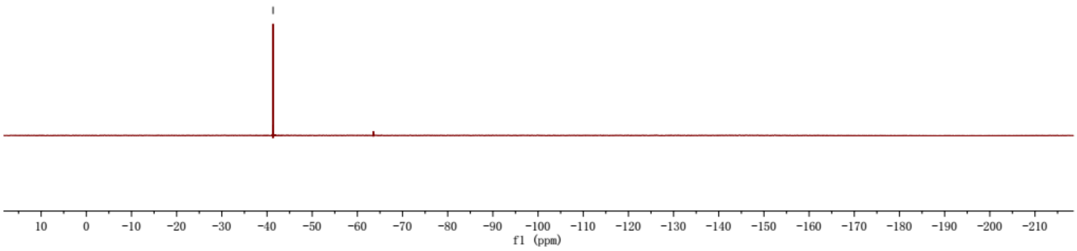

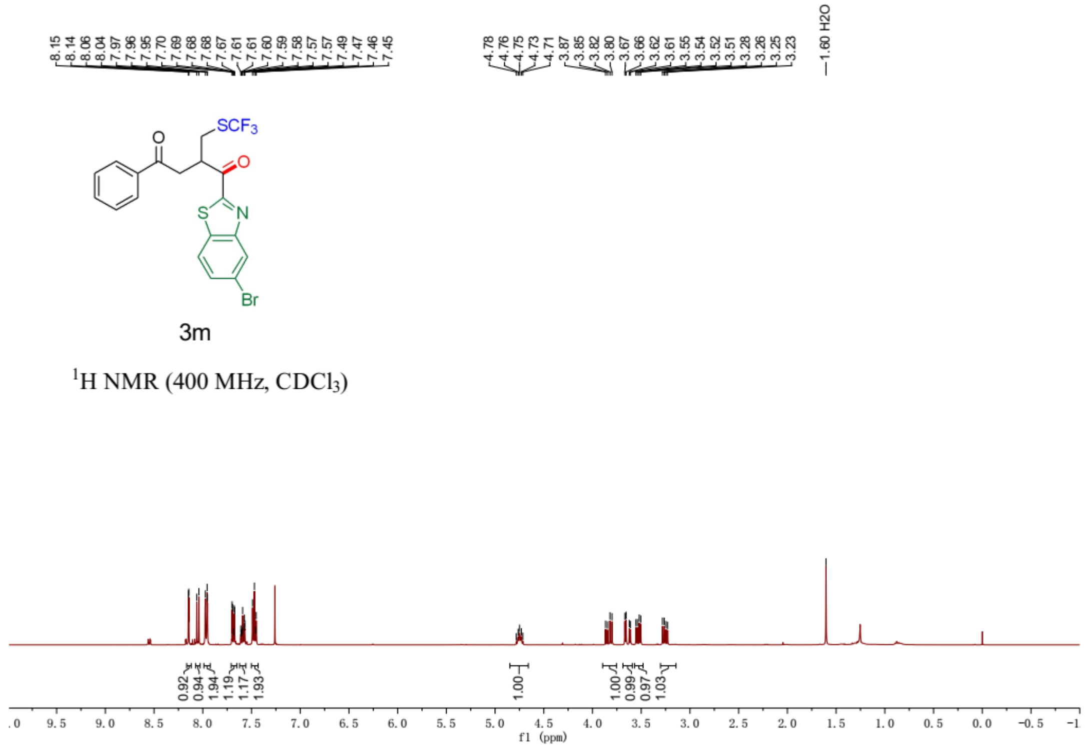

3m

^{1}H NMR (400 MHz, $CDCl_3$)

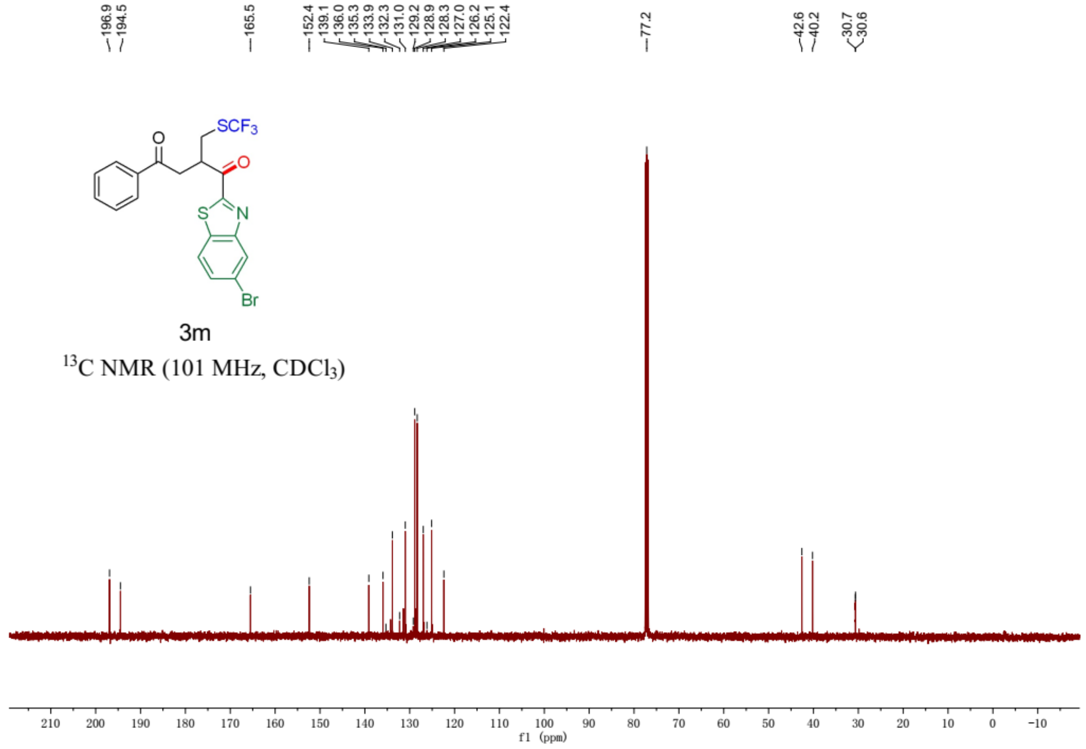

3m

^{13}C NMR (101 MHz, $CDCl_3$)

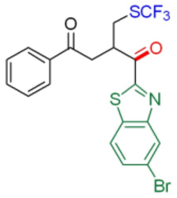

3m

^{19}F NMR (376 MHz, $CDCl_3$)

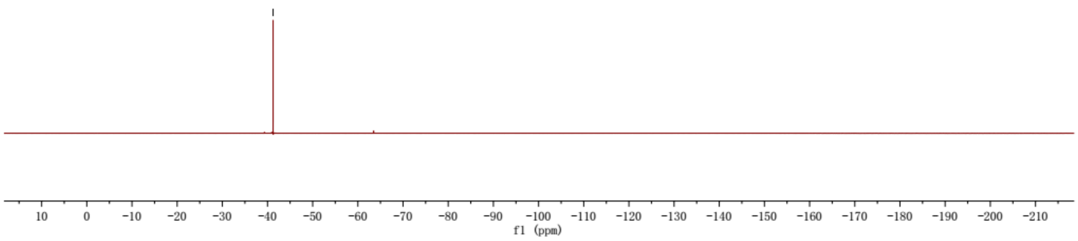

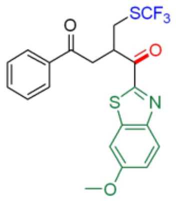

3n

^{1}H NMR (400 MHz, $CDCl_3$)

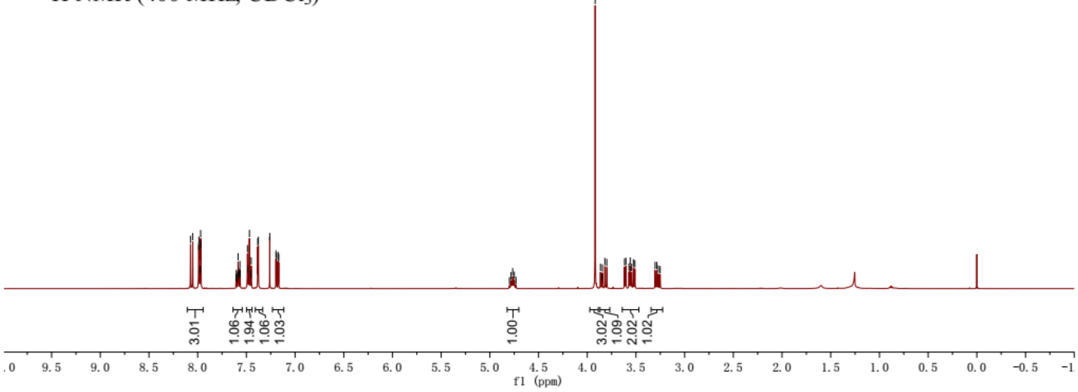

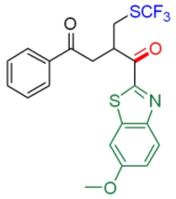

3n

^{13}C NMR (101 MHz, $CDCl_3$)

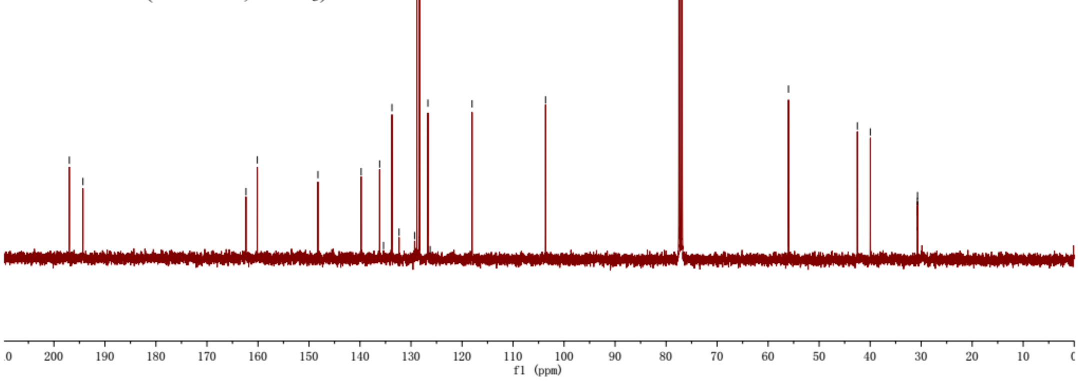

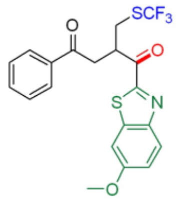

3n

^{19}F NMR (376 MHz, $CDCl_3$)

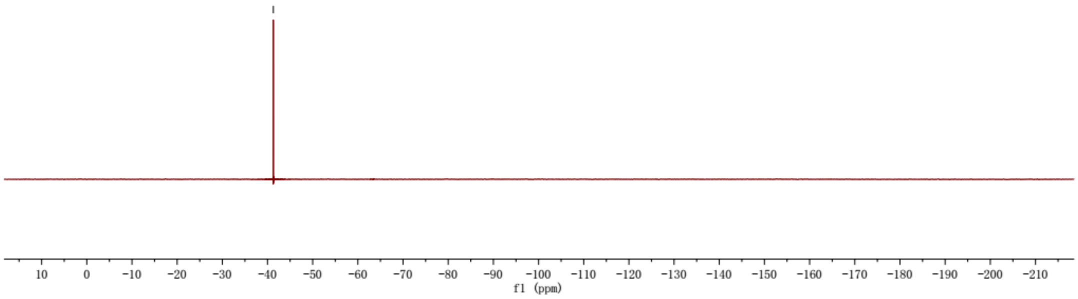

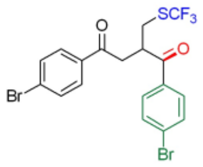

3o

^{1}H NMR (400 MHz, $CDCl_3$)

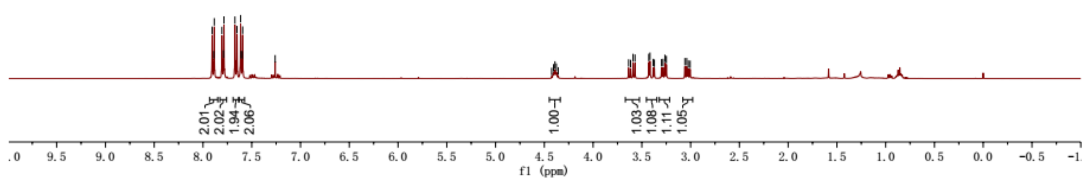

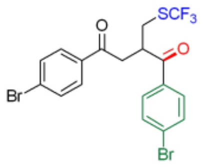

3o

^{13}C NMR (101 MHz, $CDCl_3$)

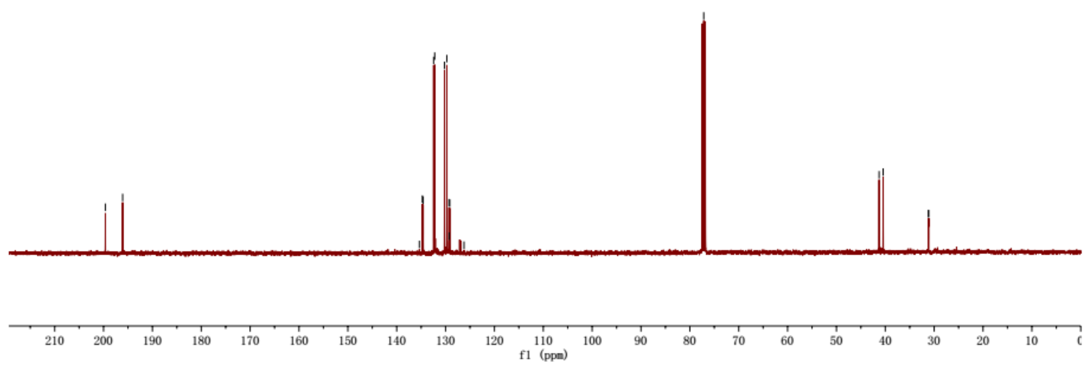

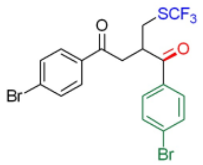

3o

^{19}F NMR (376 MHz, $CDCl_3$)

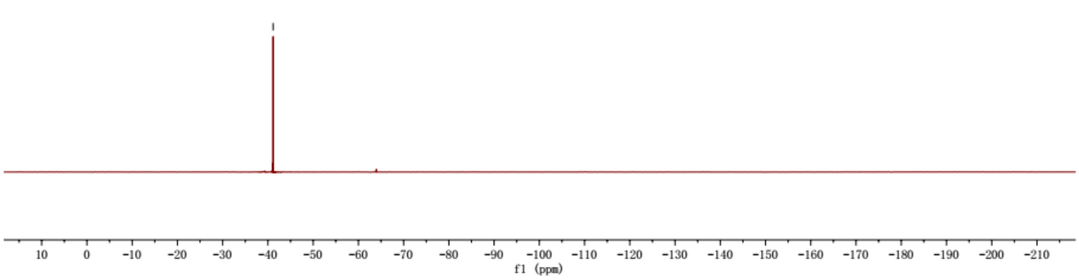

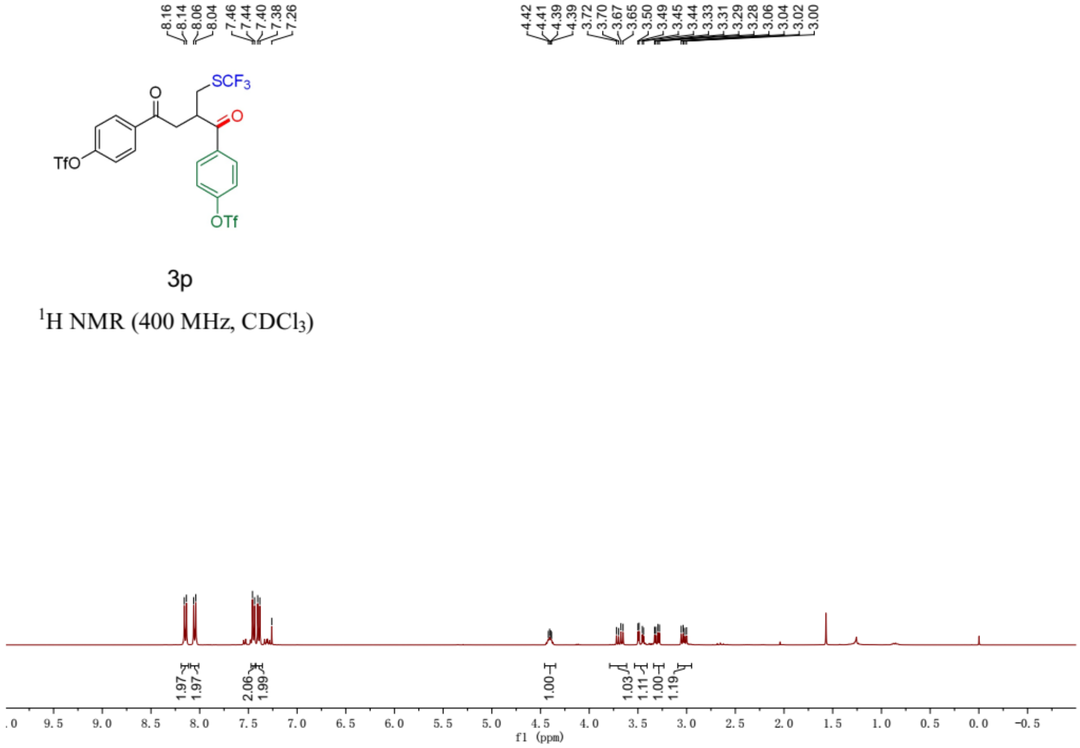

3p

^{1}H NMR (400 MHz, $CDCl_3$)

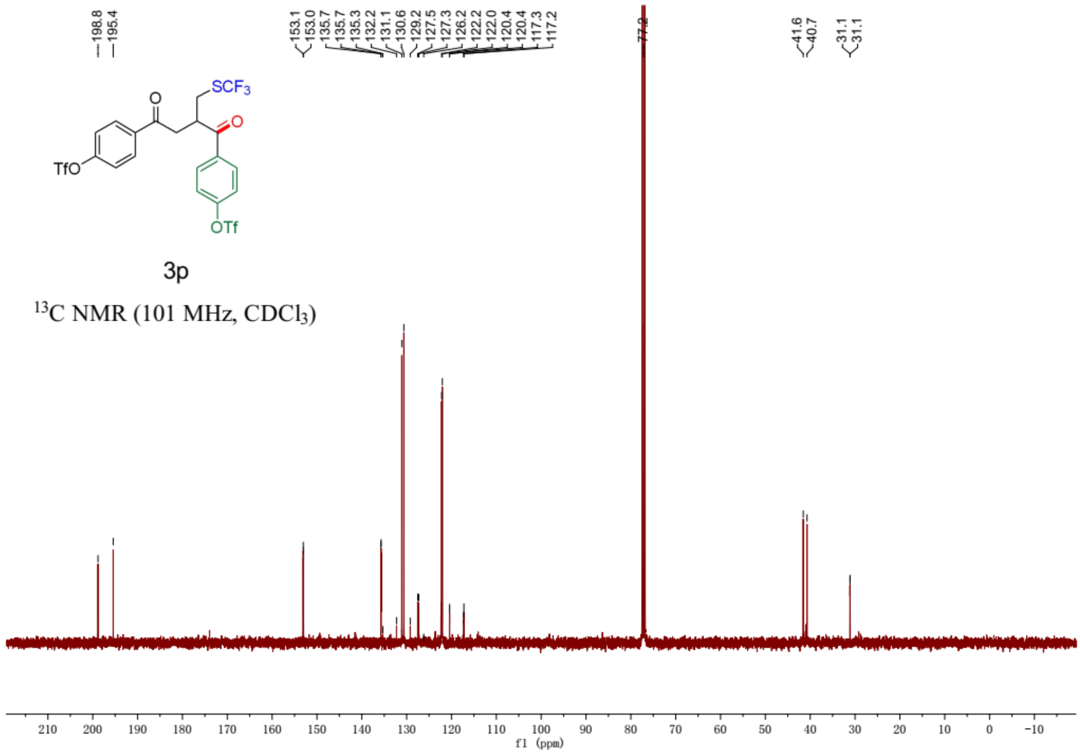

3p

^{13}C NMR (101 MHz, $CDCl_3$)

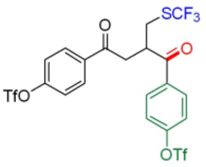

3p

^{19}F NMR (376 MHz, $CDCl_3$)

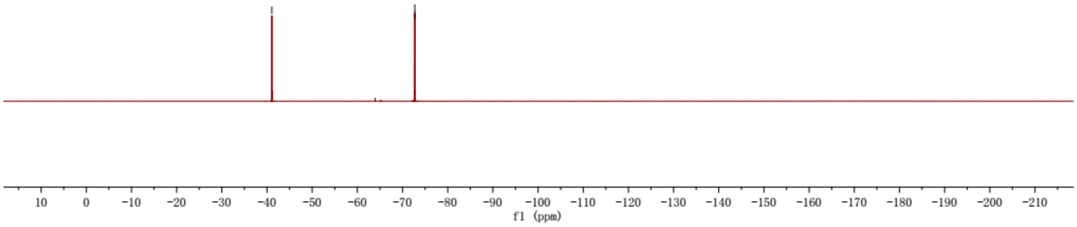

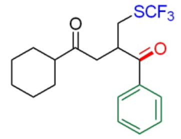

3q

^{1}H NMR (400 MHz, $CDCl_3$)

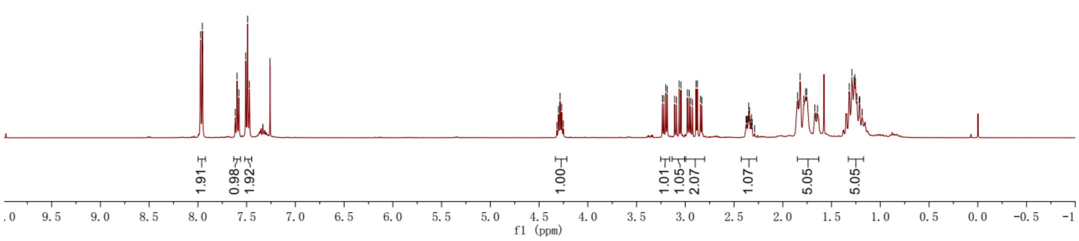

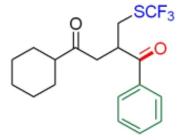

3q

^{13}C NMR (101 MHz, $CDCl_3$)

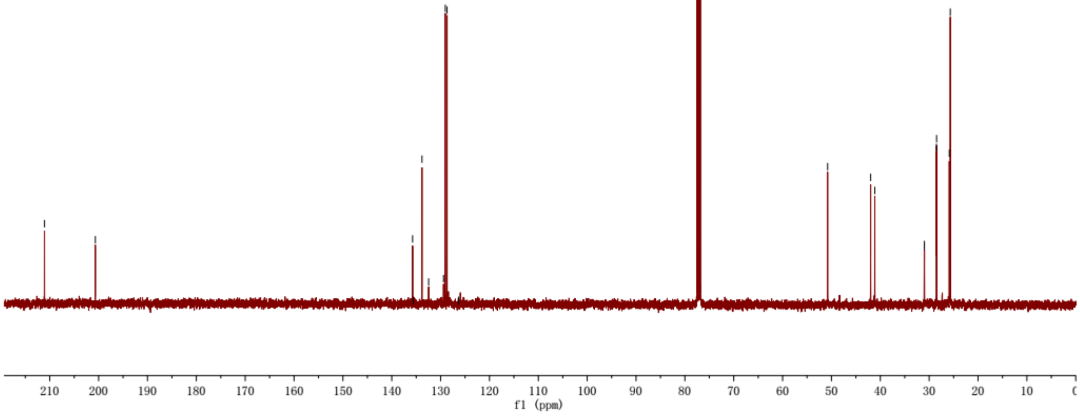

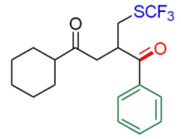

3q

^{19}F NMR (376 MHz, $CDCl_3$)

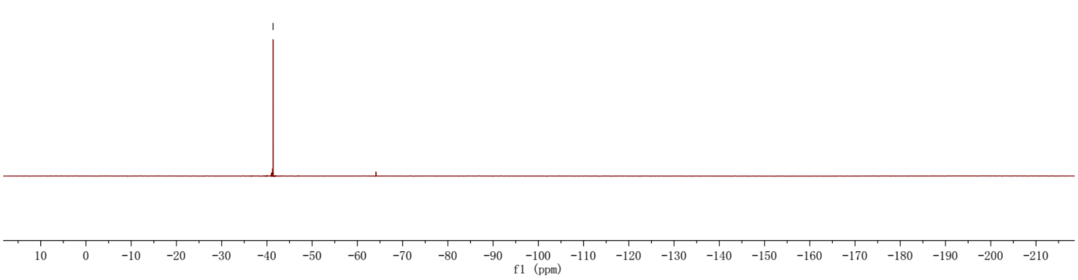

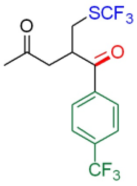

3r

^{1}H NMR (400 MHz, $CDCl_3$)

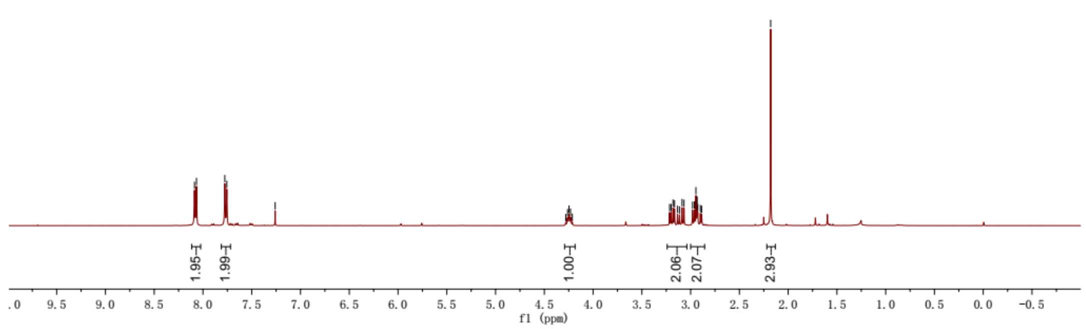

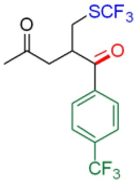

3r

^{13}C NMR (101 MHz, $CDCl_3$)

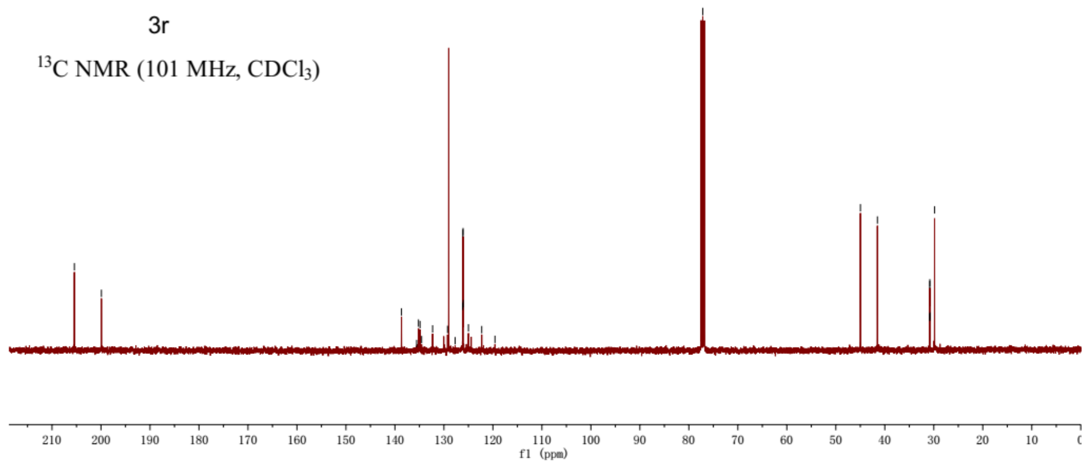

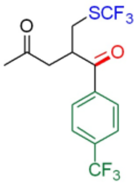

3r

^{19}F NMR (376 MHz, $CDCl_3$)

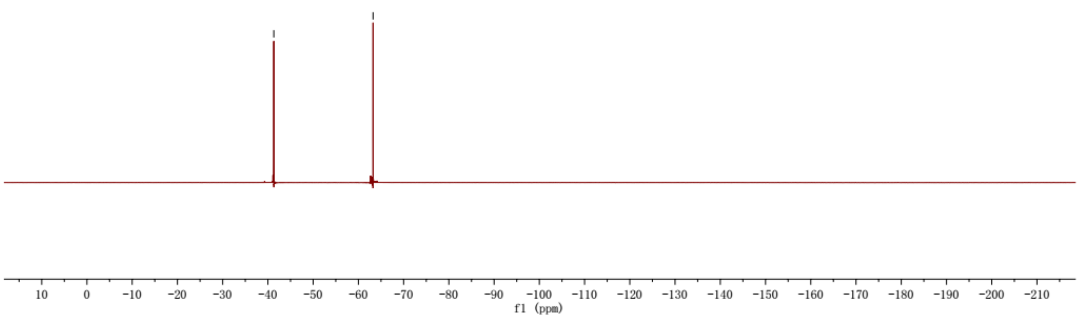

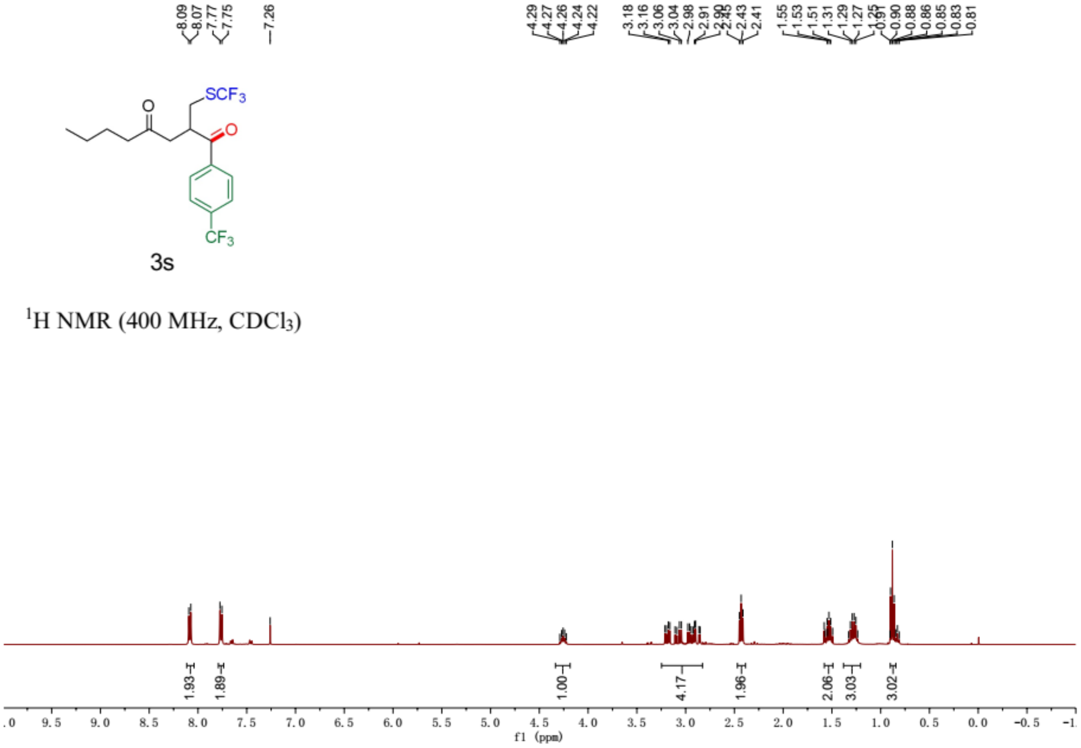

3s

^{1}H NMR (400 MHz, $CDCl_3$)

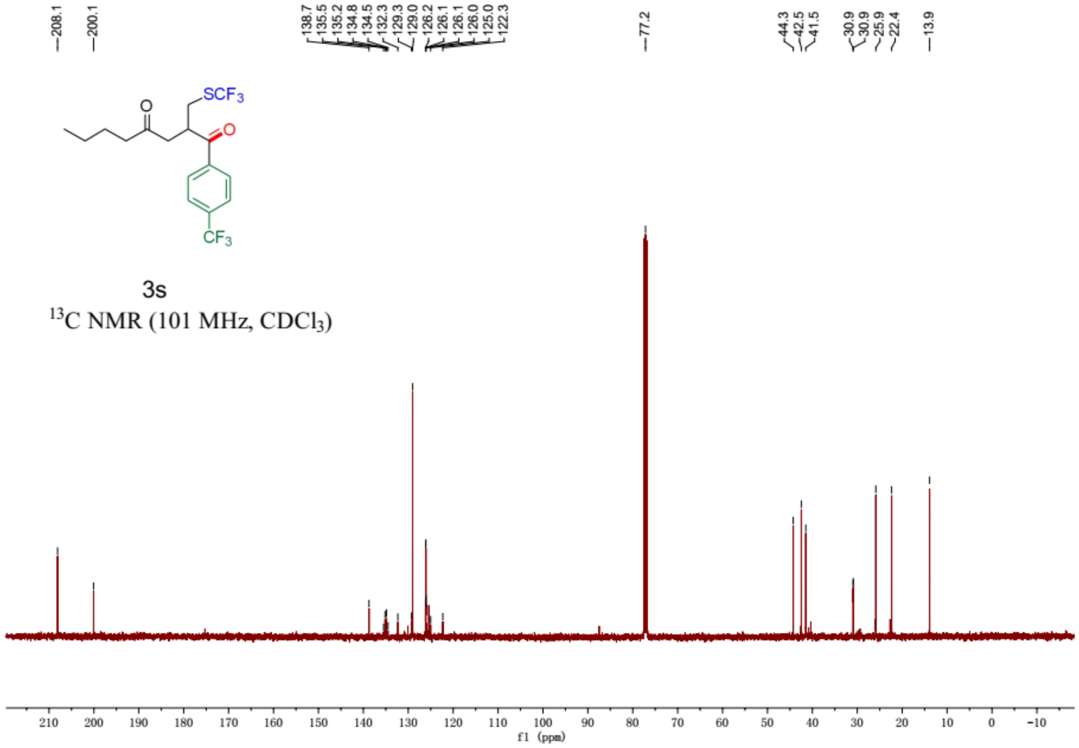

3s

^{13}C NMR (101 MHz, $CDCl_3$)

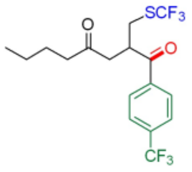

3s

^{19}F NMR (376 MHz, $CDCl_3$)

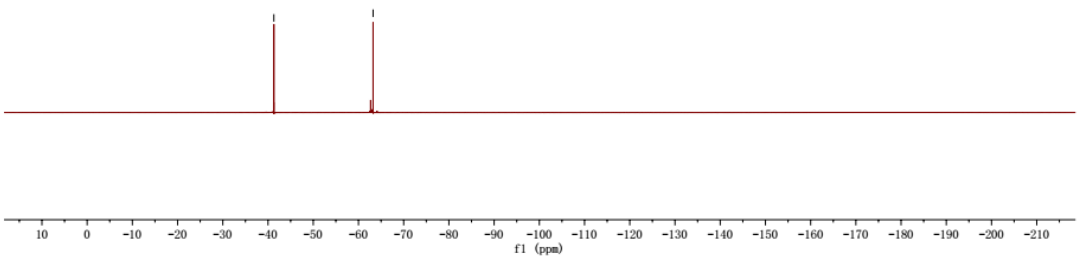

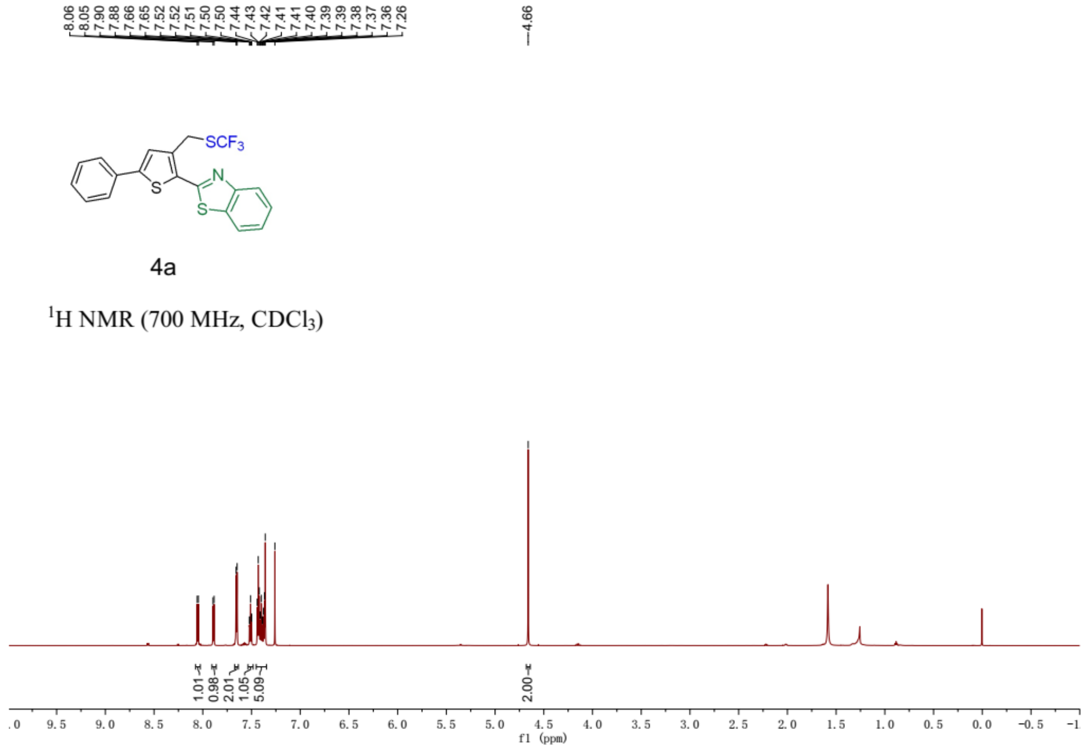
4a
^{1}H NMR (700 MHz, CDCl$_3$)

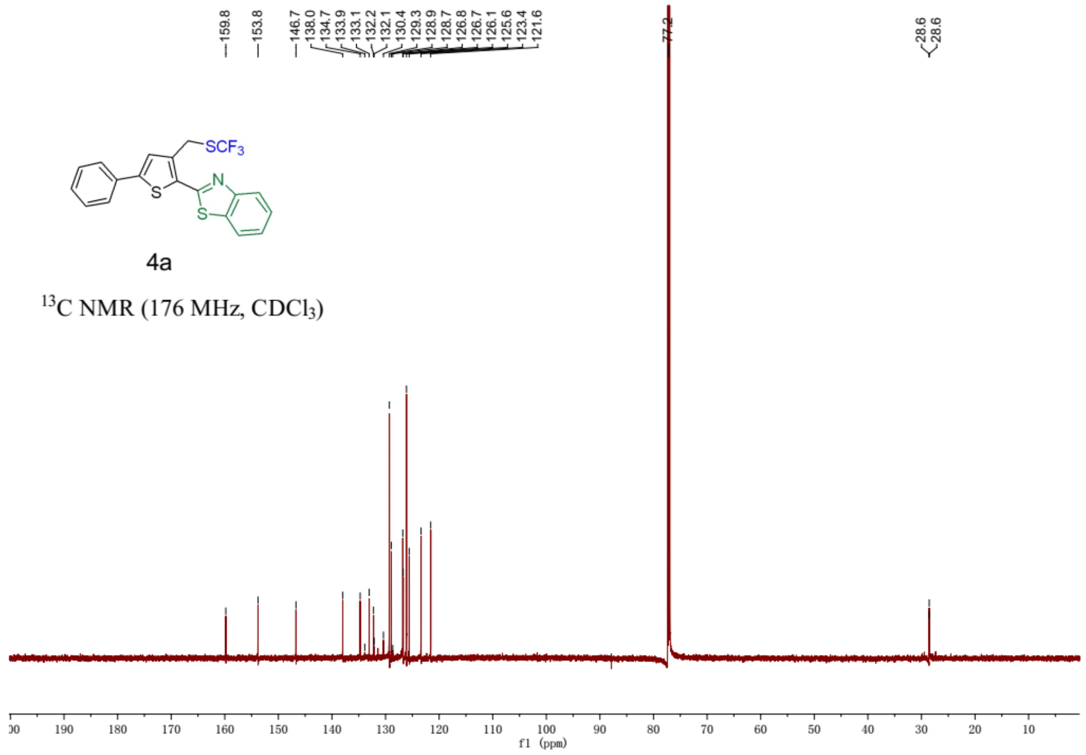
4a
^{13}C NMR (176 MHz, CDCl$_3$)

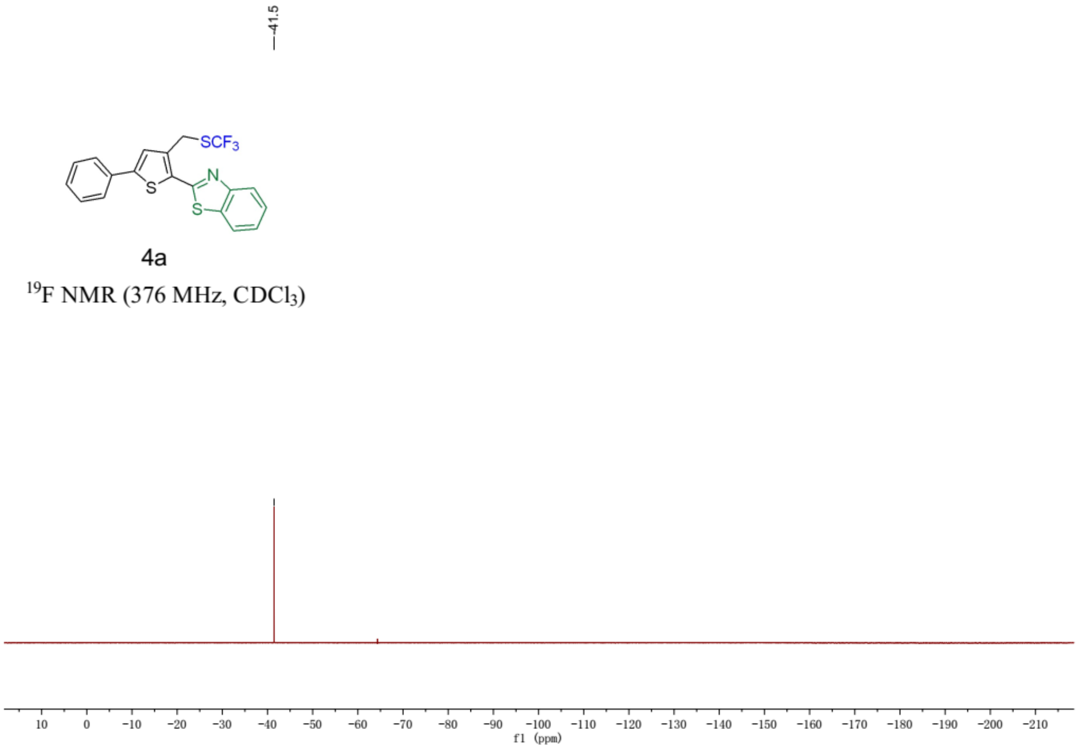
—41.5
SCF_3
N
S
S
4a
^{19}F NMR (376 MHz, $CDCl_3$)
10 0 −10 −20 −30 −40 −50 −60 −70 −80 −90 −100 −110 −120 −130 −140 −150 −160 −170 −180 −190 −200 −210
f1 (ppm)
